# Evaluation of the serum metabolome of patients with alkaptonuria before and after two years of treatment with nitisinone using LC‐QTOF‐MS

**DOI:** 10.1002/jmd2.12042

**Published:** 2019-05-31

**Authors:** Andrew S. Davison, Brendan P. Norman, Gordon A. Ross, Andrew T. Hughes, Milad Khedr, Anna M. Milan, James A. Gallagher, Lakshminarayan R. Ranganath

**Affiliations:** ^1^ Department of Clinical Biochemistry and Metabolic Medicine, Liverpool Clinical Laboratories Royal Liverpool University Hospitals Trust Liverpool UK; ^2^ Musculoskeletal Biology I, Institute of Ageing and Chronic Disease University of Liverpool, Liverpool Health Partners Liverpool UK; ^3^ Agilent Technologies Cheadle UK

**Keywords:** alkaptonuria, metabolomics, nitisinone, tyrosine

## Abstract

**Background:**

The homogentisic acid‐lowering therapy nitisinone is being evaluated for the treatment of alkaptonuria (AKU) at the National Centre for AKU. Beyond hypertyrosinemia, the wider metabolic consequences of its use are largely unknown. The aim of this work was to evaluate the impact of nitisinone on the serum metabolome of patients with AKU after 12 and 24 months of treatment.

**Methods:**

Deproteinized serum from 25 patients with AKU (mean age[±SD] 51.1 ± 14.9 years, 12 male) was analyzed using the 1290 Infinity II liquid chromatography system coupled to a 6550 quadrupole time‐of‐flight mass spectrometry (Agilent, UK). Raw data were processed using a batch targeted feature extraction algorithm and an accurate mass retention time database containing 469 intermediary metabolites (MW 72‐785). Matched entities (±10 ppm theoretical accurate mass and ±0.3 minutes retention time window) were filtered based on their frequency and variability (<25% CV) in group quality control samples, and repeated measures statistical significance analysis with Benjamini‐Hochberg false discovery rate adjustment was used to assess changes in metabolite abundance.

**Results:**

Eight metabolites increased in abundance (log_2_ fold change [FC] 2.1‐15.2, *P* < .05); 7 of 8 entities were related to tyrosine metabolism, and 13 decreased in abundance (log_2_ FC 1.5‐15.5, *P* < .05); including entities related to tyrosine (n = 2), tryptophan (n = 3), xanthine (n = 2), and citric acid cycle metabolism (n = 2).

**Conclusions:**

Evaluation of the serum metabolome of patients with AKU showed a significant difference in the abundance of several metabolites following treatment with nitisinone, including a number that have not been previously reported; several of these were not related to the tyrosine metabolic pathway.

**Synopsis:**

Nitisinone therapy has a significant impact on several metabolites beyond the tyrosine metabolic pathway, several of which appear to be related to the redox state of the cell.

**Abbreviations**
3‐(4‐HPLA): 3‐(4‐hydroxyphenyl)lactic acid
3‐(4‐HPPA): 3‐(4‐hydroxyphenyl)pyruvic acid
4‐HBA: 4‐hydroxybenzaldehyde
4‐HPA: 4‐hydroxyphenylacetate
AKU: alkaptonuria
AMRT: accurate mass retention time
FAH: fumarylacetoacetate hydrolase
HGA: homogentisic acid
HGD: homogentisate‐1,2‐dioxygenase
HPPD: hydroxyphenylpyruvic acid dioxygenase
HT1: hereditary tyrosinaemia type 1
I‐3‐L: indole‐3‐lactate
I‐3‐P: indole‐3‐pyruvate
MPP: mass profiler professional
MS/MS: tandem mass spectrometry
NAC: National Alkaptonuria Centre
QC: quality control

## 1 INTRODUCTION

Alkaptonuria (AKU, OMIM: 203500) is a rare autosomal recessive disorder of tyrosine metabolism resulting from a defect in homogentisate‐1,2‐dioxygenase (HGD, E.C.1.12.11.5), which leads to a marked increase in the circulating concentration of homogentisic acid (HGA).^1^ The pathological hallmark of AKU is “ochronosis,” which is a consequence of the deposition of a dark pigment in connective tissue, mainly cartilage, which alters its physicomechanical properties. The exact composition and structure of this pigment is unknown, but is known to result from the accumulation of HGA (Figure S1).^2^

Treatment options for AKU are conservative in large focusing on supportive and palliative measures.^2^ The HGA lowering agent nitisinone (Figure S1) has been shown to completely prevent ochronosis in AKU mice^3^ and is being evaluated as a potential treatment in AKU patients^1^, ^4^, ^5^, ^6^, ^7^, ^8^; it is however not without its own challenges as it is well documented to result in significant hypertyrosinemia in AKU^1^, ^4^, ^5^, ^6^, ^7^, ^8^, ^9^, ^10^ and hereditary tyrosinaemia type 1 (HT1, OMIM: 276700).^11^, ^12^, ^13^ The consequences of this are largely unknown in AKU; in HT1 it has been suggested this may contribute to the neurodevelopmental delay that is frequently observed in children treated with nitisinone.^14^ Several mechanisms have been proposed for this including altered metabolism of the monoamine neurotransmitters.^15^ Davison et al.^16^, ^17^ demonstrated in a cohort of AKU patients that nitisinone therapy resulted in altered urinary excretion of dopaminergic and serotoninergic neurotransmitter metabolites. However, these findings are limited as they are not a direct reflection of neurotransmitter metabolism in the central nervous system. A recent study in an animal model of HT1 suggested that the disease itself and not treatment with nitisinone is likely to be responsible for slower learning and altered behavior in mice.^18^

Recently, changes in the urine metabolome of an AKU mouse model and patients with AKU treated with nitisinone were reported.^19^ This study demonstrated novel changes in the tyrosine metabolic pathway, and unexpectedly in tryptophan and purine metabolism. While these changes in the urine metabolome provide insight into how nitisinone alters metabolism, one may postulate that changes observed in the serum are more relevant as they are a more direct reflection of internal homeostasis. Gertsman et al.^20^ reported on the impact of nitisinone therapy on the serum metabolome of patients with AKU. In addition to the expected decrease in HGA and increase in tyrosine, significant increases in acetyl‐ and γ‐glutamyltyrosine were also observed. In a separate publication^21^ in the same cohort of patients, novel disturbances in tryptophan metabolism were reported.

Herein for the first time we report the impact of nitisinone (2 mg daily) therapy on the serum metabolome in the largest cohort of AKU patients to date over a 2‐year period at the National AKU Centre (NAC) in the United Kingdom.

## 2 MATERIALS AND METHODS

### 2.1 Reagents

Water for mobile phases was purified in‐house (DIRECT‐Q 3UV Millipore water purification system). Methanol, acetonitrile, and isopropanol were purchased from Sigma‐Aldrich (Dorset, UK). Formic acid and ammonium formate were obtained from Biosolve (Netherlands) and Fisher Scientific (Germany), respectively. All reagents were liquid chromatography (LC)‐mass spectrometry (MS) grade. Acetyl‐tyrosine was purchased from Sigma‐Aldrich, UK.

## 3 PATIENTS AND SERUM SAMPLE COLLECTION

### 3.1 Ethical approval

Data collection and sample analyses at the NAC have approval from the Royal Liverpool and Broadgreen University Hospital Trusts Audit Committee (Audit no. ACO3836), this approval includes the use of patient data and biological material for metabolomics evaluation. As data and samples were collected as part of the clinical service, ethical approval was not required. Patients are informed verbally and through patient information leaflets about the clinical and research activities of the NAC and are informed that data may be used for publication. For a detailed protocol see Milan et al.^5^

### 3.2 Sample collection

Serum samples (S‐monovette; Sarstedt, Germany) were collected from patients after an overnight fast (≥8 hours). Patients' dietary intake of protein was managed through a 7‐day food diary by a combination of lower protein in diet and phenylalanine/tyrosine‐free meal exchanges. Samples were centrifuged at 1500*g* for 10 minutes at 4°C; and then deproteinized with perchloric acid (60% 5.8 M; 1:10, perchloric acid:serum), vortexed and centrifuged at 1500*g* for 10 minutes. Supernatant was stored at −20°C until analysis.

## 4 PATIENT AND QUALITY CONTROL SAMPLE PREPARATION

Patient samples were prepared by diluting 150 μL of serum with 450 μL of deionized water (DIRECT‐Q 3UV Millipore water purification system). Diluted samples were then transferred into a 96‐well plate which was then agitated on a plate shaker (MTS 2/4 m IKA, Germany) at 600 rpm for 10 minutes.

Patient group quality control (QC) samples were produced by adding 50 μL of each patient sample into a single pool. In total four group QC pools were made ([a] baseline, [b] 12‐months, [c] 24‐months, and [d] overall—containing serum from all patients and visits; this acted as a system QC). QC samples were prepared as per patient samples.

The analytical sequence of samples was performed as per published guidance.^22^ Each run commenced with 20 replicate injections of the overall pooled sample to condition the system. The order of individual samples was randomized computationally. Pooled samples were interspersed throughout the analytical sequence every tenth injection.

## 5 ANALYTICAL METHOD

### 5.1 Chromatographic conditions

Liquid chromatography was performed on an Agilent 1290 Infinity II LC system. An Atlantis dC_18_ column (3.0 × 100 mm, 3 μm, Waters, UK) was maintained at 60°C with a flow rate of 0.4 mL/min. Mobile phases were (A) water and (B) methanol both containing 5 mmol/L ammonium formate and 0.1% formic acid. The elution gradient started at 5% mobile phase B at 0‐1 minute increasing linearly to 100% B by 12 minutes, held at 100% B until 14 minutes, returning to 95% A for 5 minutes to recondition the column. Injection volume was 1 μL.

### 5.2 Quadrupole time‐of‐flight MS conditions

An Agilent 6550 quadrupole time‐of‐flight MS (QTOF‐MS) equipped with a dual jet stream electrospray ionization source was operated in 2 GHz mode, over the mass range of 50‐1700, in negative and positive polarities. A reference mass correction solution was continually infused at a flow rate of 0.5 mL/min via an external isocratic pump (Agilent, UK) for constant mass correction (see Supporting Information 1 for additional details of QTOF‐MS operating parameters and composition of reference ion solution).

## 6 METABOLITE IDENTIFICATION, DATA QC, AND STATISTICAL ANALYSES

Metabolite identification was carried out using an established accurate mass retention time (AMRT) database to match chemical entities.^19^ The database included theoretical accurate mass, measured retention time, and empirical formula. This was modified to include acetyl‐tyrosine, γ‐glutamyltyrosine, and indole‐3‐lactate (I‐3‐L). AMRT data for acetyl‐tyrosine were verified following the analysis of an analytical standard. AMRT data for γ‐glutamyltyrosine and I‐3‐L were based on the elution time associated with theoretical monoisotopic mass of each compound and were not verified using an analytical standard. Data and statistical analyses were performed using the MassHunter software suite (Agilent, UK). For additional details on data and statistical analyses, see Supporting Information 1.

## 7 RESULTS

Twenty‐five patients (13 female, mean age[±SD] 55.3[15.3] years [range 22‐75]; 12 male, mean age 44.2[15.8] years [range 22‐70]) were included at baseline and after treatment with nitisinone at 12 and 24 months.

**Figure 1:**
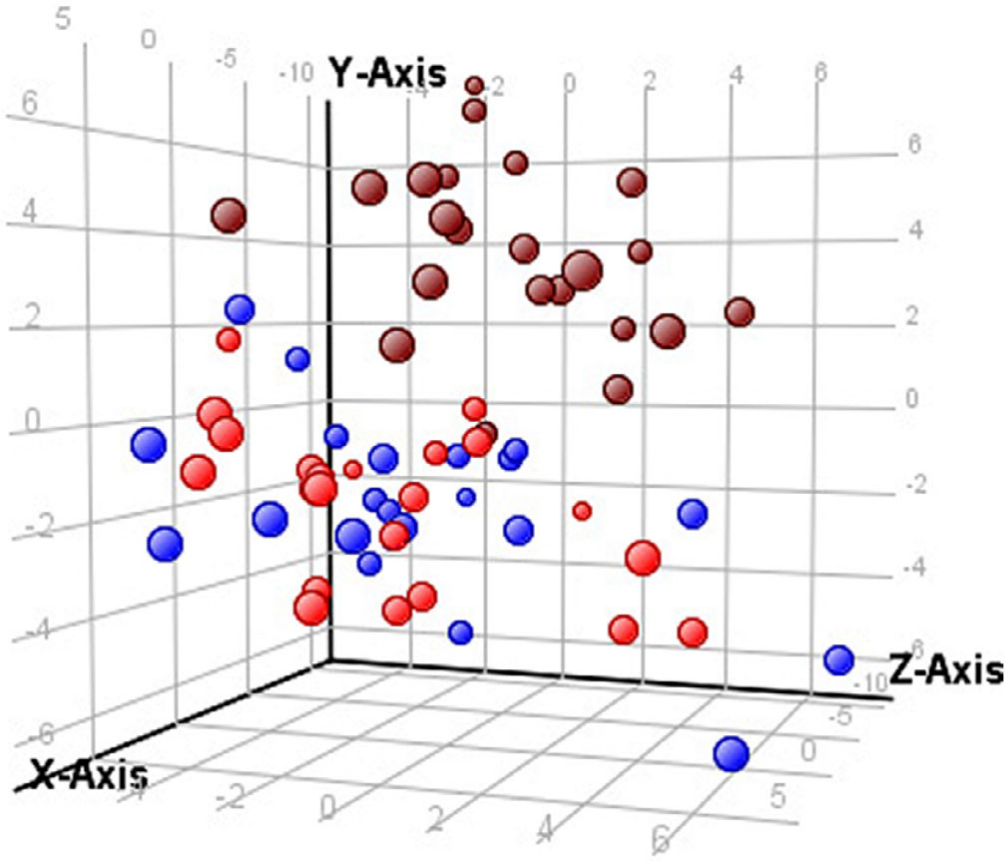
Principal component analysis of raw data from LC‐QTOF‐MS profiling experiments of serum samples from patients at baseline (pre‐nitisinone, brown circle), 12‐months (2 mg daily nitisinone, red circle), and 24‐months (2 mg daily nitisinone, blue circle) *x*‐axis—component 1, 22.09%; *y*‐axis—component 2, 9.66%; *z*‐axis—component 3, 7.78%. LC, liquid chromatography, QTOF‐MS, quadrupole time‐of‐flight mass spectrometry

Raw data from LC‐QTOF‐MS analysis showed that retention time and accurate mass ranges were 1.15‐13.93 minutes and 75.0318‐730.5955 Da, in positive and 1.03‐14.67 minutes and 75.0318‐722.6247 Da in negative polarity, respectively. Principal components analysis (Figure 1) showed clear separation between the AMRT‐matched profiles of AKU patients pre‐ vs post‐nitisinone therapy.

One hundred fifty‐one of 469 and 249 of 469 metabolites were aligned across all samples in positive and negative polarities (for matched compounds see Table S2), respectively, at baseline, and after nitisinone treatment at 12 and 24 months. After filtering entities based on their frequency and variability across replicate injections of pooled QC samples from each experimental group, 123 and 209 entities were retained from positive and negative polarity profiling experiments, respectively. Of these, 60 and 121, respectively, were shown to be significantly different (*P* < .05) following nitisinone therapy. Those with a log_2_ fold change (FC) >2 at 12 and/or 24 months are summarized in Table 1. Applying this cut‐off, eight (6.5%) entities were considered to increase in abundance and 13 (10.7%) decrease in abundance.

**Table 1:** Serum metabolite changes identified post‐nitisinone therapy at 12 and 24 months in patients with AKU using an in‐house AMRT database

Compound	Log_2_ FC		*P* value	Abundance		Metabolic process/pathway affected
	12‐Months	24‐Months		Down	Up	
Glycocholate	12.7	12.7	<.001	√		Bile acid
Succinic acid	11.4	11.4	<.001	√		Citric acid cycle
α‐Ketoglutaric acid	10.0	10.0	<.001	√		Citric acid cycle
Trans‐4‐hydroxyproline	15.5	15.5	<.001	√		Collagen
Homoserine	12.5	12.5	<.001	√		Methionine/cysteine
Mevalolactone	10.7	10.7	<.001	√		Steroid
l‐Allothreonine	11.8	11.8	.02	√		Threonine
Trigonelline	12.8	12.8	<.001	√		Tryptophan
4‐Quinolinecarboxylic acid	8.3	8.3	<.001	√		Tryptophan
Indole‐3‐lactate	2.1	2.3	<.001		√	Tryptophan
4‐Hydroxyphenylacetate	4.2	5.4	<.001		√	Tyrosine
Benzaldehyde	6.9	6.9	<.001	√		Tyrosine
Homogentisate	4.0	5.0	<.001	√		Tyrosine
4‐Hydroxybenzaldehyde	14.4	14.4	<.001		√	Tyrosine
l‐*N*‐Acetyl‐tyrosine	15.5	15.2	<.001		√	Tyrosine
γ‐l‐Glutamyl‐l‐tyrosine	3.2	3.0	<.001		√	Tyrosine
3‐(4‐Hydroxyphenyl)lactate	6.4	6.4	<.001		√	Tyrosine
l‐Tyrosine	2.7	2.7	<.001		√	Tyrosine
Mandelic acid	12.6	12.0	<.001		√	Tyrosine
Inosine	10.8	10.8	<.001	√		Purine
Uridine	2.1	1.5	<.001	√		Pyrimidine

*Note*: Abundance expressed as log_2_ FC compared to baseline (pre‐nitisinone treatment). Log_2_ FC included if >2 at 12‐ and or 24‐months.

Abbreviation: AMRT, accurate mass retention time.

Nine (43%) of the 21 metabolites that were affected following treatment with nitisinone relate to tyrosine metabolism. Many of the other metabolites that had altered abundance following nitisinone therapy did not follow a clear theme apart from tryptophan, citric acid cycle, and purine/pyrimidine metabolism.

## 8 DISCUSSION

Over the last two decades, there have been several reports on the use of nitisinone to treat AKU. Its inhibition of HPPD (Figure S1) has been shown to dramatically reduce the circulating concentration of HGA,^1^, ^4^, ^5^, ^6^, ^7^, ^8^ but leads to marked hypertyrosinemia.^1^, ^4^, ^5^, ^6^, ^7^, ^8^, ^9^, ^10^ Beyond hypertyrosinemia, there is very little reported on the biochemical consequences of nitisinone therapy. Herein, we report the impact of nitisinone therapy on the serum metabolome, using LC‐QTOF‐MS and a validated strategy to identify metabolites using an AMRT database developed in‐house.^19^ This study is unique as it includes the largest cohort of patients with AKU to date, taking a 2 mg daily dose of nitisinone over 24 months. In addition, as it is based on the analysis of serum it is a better reflection of the impact of nitisinone on internal homeostasis.

This study confirms previous reports that nitisinone treatment in AKU results in a marked reduction in serum HGA and increase in tyrosine. In addition, a significant increase in 3‐(4‐hydroxyphenyl)lactate (3‐(4‐HPLA)) was observed. Unexpectedly 3‐(4‐hydroxyphenyl)pyruvate (3‐(4‐HPPA)), the metabolite immediately proximal to the site of action of nitisinone, was not increased (Figure S1). The marked increase in HPLA is an expected consequence of nitisinone therapy, but has not been reported in serum. There is an equilibrium between 3‐(4‐HPPA) and 3‐(4‐HPLA) (Figure S1) and it is likely that the reason for only observing significant increases in latter is that the sample pH shifted the equilibrium position to favor its formation. This pattern has been previously reported^19^ in a study that reported on the urine metabolome of patients with AKU treated with nitisinone; a 84‐ and 3.3‐fold (raw FC) increase in 3‐(4‐HPLA) and 3‐(4‐HPPA) were reported, respectively.

Marked increases in the tyrosine conjugates acetyl‐tyrosine and γ‐glutamyltyrosine were also observed. The log_2_ FC observed was very similar for tyrosine and γ‐glutamyltyrosine, but that of acetyl‐tyrosine was markedly higher. The latter suggests that an equilibrium shift between tyrosine and acetyl‐tyrosine may exist, but not for tyrosine and γ‐glutamyltyrosine. Norman et al.^19^ showed that urinary tyrosine was significantly higher than acetyl‐tyrosine and γ‐glutamyltyrosine in a cohort of AKU patients on 2 mg daily of nitisinone supporting that an equilibrium shift between tyrosine and tyrosine conjugates exists. In contrast, Gertsman et al.^20^ reported a proportional increase in plasma tyrosine, acetyl‐tyrosine and γ‐glutamyltyrosine following nitisinone suggesting there is no equilibrium shift. While direct comparisons between the magnitudes of FC cannot be made as herein we report log_2_ FC, the proportions of metabolites are clearly different between the two studies. This may be due to the small number of patients in the study reported by Gertsman et al.^20^ and that a 2 mg dose was used only in our study. Nonetheless, our findings support that nontraditional metabolic pathways are active in the face of tyrosine excess.

**Figure 2:**
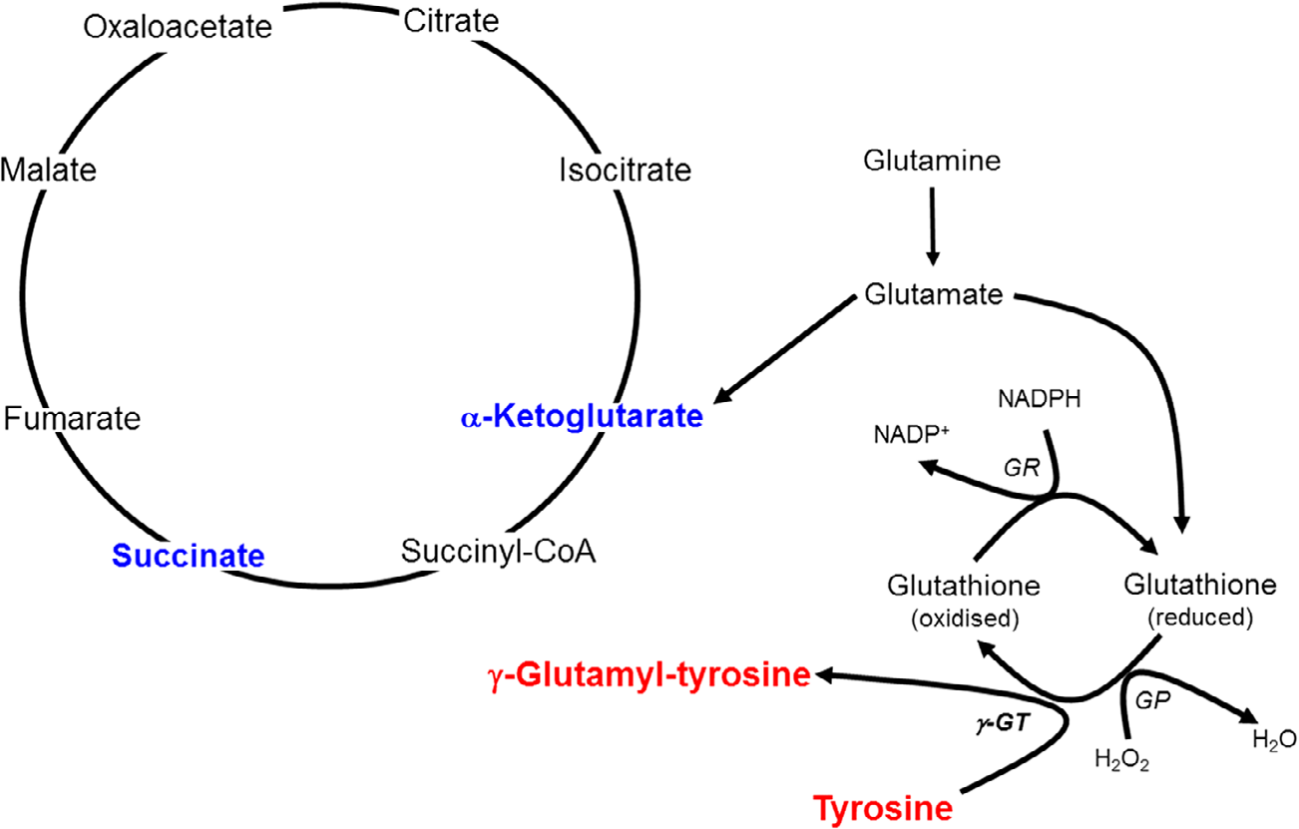
Proposed mechanism for the formation of γ‐glutamyltyrosine in AKU following nitisinone therapy. Entities in red and blue represent an increase and decrease in metabolite abundance, respectively. AKU, alkaptonuria; GP, glutathione peroxidase; GR, glutathione reductase; GT, γ‐glutamyltranspeptidase

The elevated γ‐glutamyltyrosine suggests that glutathione metabolism and the redox state of cell^23^, ^24^ are altered following treatment with nitisinone. This is of particular importance to AKU as elevated HGA is thought to lead to a pro‐oxidant environment where “soluble melanins” are formed.^25^ Increasing evidence to support HGA‐induced oxidative stress has been reported in vitro in serum^26^ and cellular models,^27^ and in patients with AKU.^28^ For a recent review on oxidative stress and its contribution to the mechanisms of the ochronotic process, see Ref. 29. One may postulate that treatment with nitisinone reduces the burden on the glutathione cycle improving glutathione availability. In turn, this may enable transfer of the glutamyl moiety from glutathione to tyrosine, via the action of γ‐glutamyl‐transpeptidase^23^, ^24^ to form γ‐glutamyltyrosine. Interestingly, this study also revealed a marked decrease in the citric acid cycle metabolites α‐ketoglutaric and succinic acid, the keto acids of α‐ketoglutarate and succinate, following nitisinone. In this study, glutamine levels did not change, thus one may hypothesize that glutamate formed from glutamine is preferentially converted to glutathione and not α‐ketoglutaric and succinic acid (Figure 2). The significance of this is not understood and requires further investigation.

The significance of increased acetyl‐tyrosine is unknown, but has been reported in serum^20^ and urine^19^ of AKU patients treated with nitisinone. It has also been observed in the urine of patients with hereditary tyrosinaemia type 2 (OMIM: 276600).^30^ One may postulate that acetyl‐tyrosine represents a more efficient way to eliminate the excess tyrosine from the body as it is more water soluble than tyrosine. Interestingly acetyl‐tyrosine has also been reported as an additive to foodstuffs given to patients on nutritional support.^31^

4‐Hydroxyphenylacetate (4‐HPA), 4‐hydroxybenzaldehyde (4‐HBA), and benzaldehyde were increased significantly following treatment with nitisinone; these have not been previously reported in the serum metabolome of patients with AKU following nitisinone treatment. 4‐HPA has been reported in urine following treatment with nitisinone.^19^ 4‐HPA is generated from gut microbiota^32^; one may hypothesize that the increase observed herein resulted from less oxidative stress following nitisinone, which increased microbiotic metabolism. This is the first report in humans, but has been seen previously observed in a rat model of oxidative stress.^32^

The increase in 4‐HBA has been reported in the urine of patients with AKU following nitisinone,^19^ but the decrease in benzaldehyde has never been observed. The significance of both entities is uncertain. 4‐HBA is a naturally occurring compound that originates from the saprophytic perennial *Gastrodia elata*.^33^ Recent studies have reported the therapeutic effects of benzaldehydes in a number of areas including wound healing, cancer, vascular disease, and renal disease.^34^, ^35^, ^36^, ^37^ In the context of AKU, the significance of 4‐HBA and benzaldehyde are unknown and require further investigation. One may speculate that they relate to the benzoquinones, proposed as intermediates in the formation of ochronotic pigment observed in AKU.^19^

Mandelic acid was also increased following treatment with nitisinone, which has been reported previously.^19^ The significance of this is uncertain, but urinary elevation has previously been observed in patients with PKU on a phenylalanine‐restricted diet.^38^

The “off target effects” of any drug are essential when considering its suitability in treating a patient.^39^ Nitisinone therapy in AKU and HT1 has long been associated with altered‐tyrosine metabolism, and so can be considered a “targeted effect.” More recently off targets effects of nitisinone have been reported due to its impact on tryptophan metabolism. A decrease in 5‐hydroxyindoleacetic acid (serotonin metabolite) has been reported in the cerebral spinal fluid and urine of patients with HT1^15^ and AKU,^16^, ^17^ respectively. Serum tryptophan itself has been shown not to change following treatment with nitisinone^9^; in contrast, urinary tryptophan has been shown to decrease.^19^ These differences may be explained by the fact that tryptophan is highly protein bound (~90%‐95%)^40^ and measurement in serum reflects total tryptophan and urinary tryptophan reflects free tryptophan. The biologically active fraction is the free fraction, which is not typically measured in serum, and the majority is metabolized via the kynurenine pathway.^40^ Herein, I‐3‐L, 4‐quinolinecarboxylic acid, and trigonelline were the only tryptophan‐related metabolites that were changed following nitisinone. The significance of the latter two metabolites is uncertain, but reinforces that downstream metabolism of kynurenine and niacin may be altered following nitisinone treatment. Gertsman et al.^21^ also demonstrated an increase in plasma I‐3‐L, but additionally indole‐3‐pyruvate (I‐3‐P). It has been proposed that the increased activity in the indole pyruvate pathway results from tryptophan aminotransferase having a higher affinity for tryptophan in the presence of keto acids (eg, 4‐HPPA, 4‐HPLA).^41^ The reason for not observing I‐3‐P is uncertain, however one may speculate that I‐3‐L and not I‐3‐P was increased due to reaction conditions favoring its formation. In addition, differences may in part be explained by different approaches to sample preparation, chromatographic and MS conditions, patient cohorts, and nitisinone doses used. In addition, a separate study showed urinary xanthurenic acid, and l‐kynurenine and indoxyl sulfate were increased and decreased following nitisinone therapy, respectively.^19^ The significance of these changes is unknown.

Beyond tryptophan metabolism, there are limited reports on the off target effects of nitisinone on the metabolome. Herein, we have shown that trans‐4‐hydroxyproline decreases significantly following treatment. This is of particular relevance to AKU as it suggests that there may be decreased collagen breakdown. This requires further investigation as previous authors^42^ have demonstrated a very low cartilage turnover state in AKU patients. In contrast, a different study reported that cartilage degradation, as well as bone resorption markers were elevated in AKU patients compared to controls.^43^

In addition, significant decreases were observed in the purine and pyrimidine precursors inosine and uridine, respectively. In a previous study, decreased excretion of the purine metabolites adenine and allantoin were reported in urine from AKU patients and AKU mice treated with nitisinone.^19^ Patients included in this study and previous^19^ were not on uric acid‐lowering medication, however were on a protein‐restricted diet which may contribute to this change. A plausible explanation for the observations in serum and in urine may also relate to changes in the oxidative state of the cell. Allantoin has previously been suggested as a marker of oxidative stress,^44^ one may postulate that the decreases observed in purine and pyrimidine metabolites are a reflection of reduced oxidative stress in the face of lower HGA concentrations.^44^

Four unrelated entities were also shown to be decreased following nitisinone therapy; glycocholate, homoserine, mevalonolactone, and allothreonine. The reasons for this remain of unknown significance and require further investigation.

It is important to highlight several limitations in this study. First, while the number of patients included in this study is the largest reported to date, metabolites identified need validating in a larger cohort of patients. Moreover, an untreated group of AKU patients would provide greater credibility that the findings presented herein are a consequence of nitisinone therapy. In addition, while we believe the AMRT database used in this study is comprehensive, the targeted evaluation of the serum metabolome precludes the identification of novel changes in metabolites. Furthermore, the use of nonselective sample preparation to gain broad coverage of the metabolome may have limited metabolite detection if present at a low concentration or if changes were not reproducible due to ion suppression.

## 9 CONCLUSIONS

Evaluation of the impact of nitisinone treatment on the serum metabolome of patients with AKU revealed a number of novel changes including a number that are not directly related to the tyrosine metabolic pathway. Some of these changes can be explained by the impact of nitisinone therapy on the cellular redox state and the wider impact of metabolites on enzyme activity. Further work is required to provide greater insight into the changes observed in the serum metabolome following nitisinone therapy.

## AUTHOR CONTRIBUTIONS

A.S.D. and B.P.N. conceived, designed and performed research, analyzed data, and wrote the first draft of the article. A.M.M., G.A.R., A.T.H., M.K., J.A.G., and L.R.R. were involved in designing the research and reviewed the manuscript. A.S.D. is the guarantor and corresponding author for this work.

## CONFLICT OF INTEREST

The authors declare no potential conflict of interest.

## COMPLIANCE WITH ETHICS GUIDELINES

All procedures reported in this review were in accordance with the ethical standards of the local Hospital ethics committee and with the Helsinki Declaration of 1975, as revised in 2000. Analysis of the samples from patients attending the National Alkaptonuria Centre and all subsequent data analysis have been approved by the Royal Liverpool and Broadgreen University Hospital Trust Audit Committee (Audit no. ACO3836).

## Supporting information


**Appendix S1**: Supplementary information 1

**Figure S1** Tyrosine metabolic pathway ‐ highlighting (i) the metabolic fate of tyrosine in health, (ii) site of the enzyme defect observed in Alkaptonuria, homogentisate dioxygenase (HGD, EC 1.13.11.5) and Hereditary Tyrosinaemia type‐1, fumarylacetoacetate hydrolase (FAH, EC 3.7.1.2), and (iii) the site where nitisinone inhibits 4‐hydroxyphenylpyruvate dioxygenase (HPPD, EC 1.13.11.27) activity.

**Table S1** Summary of accurate mass and retention time data for metabolites that make up in‐house AMRT database used in this study.

**Table S2** Summary of metabolites that were aligned and matched across all samples at baseline, and after nitisinone treatment at 12‐ and 24‐months. Metabolites were aligned using Profinder software (Build 08.00, Agilent, UK); a targeted feature extraction was used to align profiled experimental data with data in the accurate mass and retention time database containing 469 intermediary metabolites. Metabolites aligned and matched in both negative (n = 249) and positive (n = 151) polarities are detailed. Feature extraction employed a window of theoretical accurate mass ± 10 ppm and retention time ± 0.15mins. Allowed species were: H^+^, Na^+^ and NH_4_^+^ for positive polarity; and H^−^ and CHO_2_^−^ for negative polarity. Dimers were allowed for both polarities. Charge state range was 1–2.
