# Supplementary material for: Evaluation of the serum metabolome of patients with alkaptonuria before and after two years of treatment with nitisinone using LC‐QTOF‐MS
Source: JIMD Rep. 2019 May 31;48(1):67–74. doi: 10.1002/jmd2.12042 (PMC6606987; doi:10.1002/jmd2.12042)
Supplement: Supplementary file 1 — Appendix S1: Supplementary information 1 [file JMD2-48-67-s001.docx]

**Supplementary information 1**

***Quadrupole time of flight mass spectrometer operating conditions***

Capillary and fragmentor voltages were 4000V and 380V, respectively. Desolvation gas temperature was 200°C with flow rate at 15L/min. The sheath gas temperature was 300°C with flow rate at 12 L/min and nebulizer pressure was 40 psig and nozzle voltage 1000V. Data acquisition rate was 3 spectra/second.

***Preparation of reference mass correction solution***

Reference mass correction solution was prepared in 95:5 methanol:water containing 5mmol/L purine (C_5_H_4_N_4_, CAS No.: 120-73-0), 100mmol/L trifluoroacetic acid ammonium salt (TFA; CF_3_CO_2_NH_4_, CAS No.: 3336-58-1) and 2.5mmol/L hexakis(1H, 1H, 3H-tetrafluoropropoxy)phosphazine (HP-0921; C_18_H_18_F_24_N_3_O_6_P_3_, CAS No.: 58943-98-9) (Agilent, Cheadle, UK). Reference ions monitored were: purine (m/z 121.0509) and HP-0921 (m/z 922.0098) (positive ionisation) and TFA (m/z 112.9856), purine (m/z 119.0363) and HP-0921 (HP-0921 + formate adduct: m/z 966.0007) (negative ionisation).

**Data acquisition and handling parameters**

Data were acquired with Data Acquisition software (Build 06.00, Agilent, UK). Quality checks and processing of raw data files (Agilent ‘.d’ files) were performed with Qualitative Analysis software (Build 07.00, Agilent, UK).

Extracted ion chromatograms of reference masses were performed to check mass accuracy remained <5 ppm throughout the run and that the reference ion signal did not drop out during the chromatographic run. In addition, to check chromatographic reproducibility binary pump pressure curves for injections across each analytical sequence were overlaid. Mass accuracy and chromatographic reproducibility were acceptable for all experiments performed.

Profiling sample data acquired were mined for signals representing AMRT database compounds using ‘targeted feature extraction’ with Profinder software (Build 08.00, Agilent, UK). Targeted feature extraction uses the molecular formulae from the AMRT database to extract and group spectral signals (i.e. adducts, isotopes and multimers) that correspond to individual database compounds. Feature extraction employed a window of theoretical accurate mass ±10ppm and database retention time ±0.3mins. Allowed species were: H^+^, Na^+^ and NH_4_^+^ (positive polarity) and H^-^ and CHO_2_^-^ (negative polarity). Dimers were allowed for both polarities. Charge state range was 1-2.

**Data quality control and statistical analysis**

Quality control filters were applied to entities obtained from targeted feature extraction in each profiling experiment. Entities were retained if present in at least two samples per experimental group. Data files were then exported from Profinder as ‘.CEF’ files as a whole batch for each profiling experiment and imported to Mass Profiler Professional (MPP) software (Build 14.5, Agilent, UK).

In MPP sample data files were baselined to the median of all samples and chemical entities were filtered based on the data from injection of QC pooled samples. Entities were first filtered based on frequency, and retained if observed in 100% of replicate injections for at least one sample group pool. Further filtering was then based on variability, and entities were retained if peak area coefficient of variation remained <25% across replicate injections for each sample group pool.

Statistical analyses performed in MPP were based on compound signal intensity, expressed as total peak area. Profiles were compared at 12 and 24 months on 2mg daily nitisinone with pre-nitisinone profiles by one-way repeated measures ANOVA with Benjamini-Hochberg false-discovery rate adjustment. Log_2_ FC was calculated pairwise between all groups within each dataset, and this was based on raw peak area data. Changes were deemed significant if asymptotic p value was <0.05. Principal components analyses employing four-component models were also performed on each filtered dataset using MPP.
