# Supplementary material for: Evaluation of the serum metabolome of patients with alkaptonuria before and after two years of treatment with nitisinone using LC‐QTOF‐MS
Source: JIMD Rep. 2019 May 31;48(1):67–74. doi: 10.1002/jmd2.12042 (PMC6606987; doi:10.1002/jmd2.12042)
Supplement: Supplementary file 2 — Figure S1 Tyrosine metabolic pathway ‐ highlighting (i) the metabolic fate of tyrosine in health, (ii) site of the enzyme defect observed in Alkaptonuria, homogentisate dioxygenase (HGD, EC 1.13.11.5) and Hereditary Tyrosinaemia type‐1, fumarylacetoacetate hydrolase (FAH, EC 3.7.1.2), and (iii) the site where nitisinone inhibits 4‐hydroxyphenylpyruvate dioxygenase (HPPD, EC 1.13.11.27) activity. [file JMD2-48-67-s002.docx]

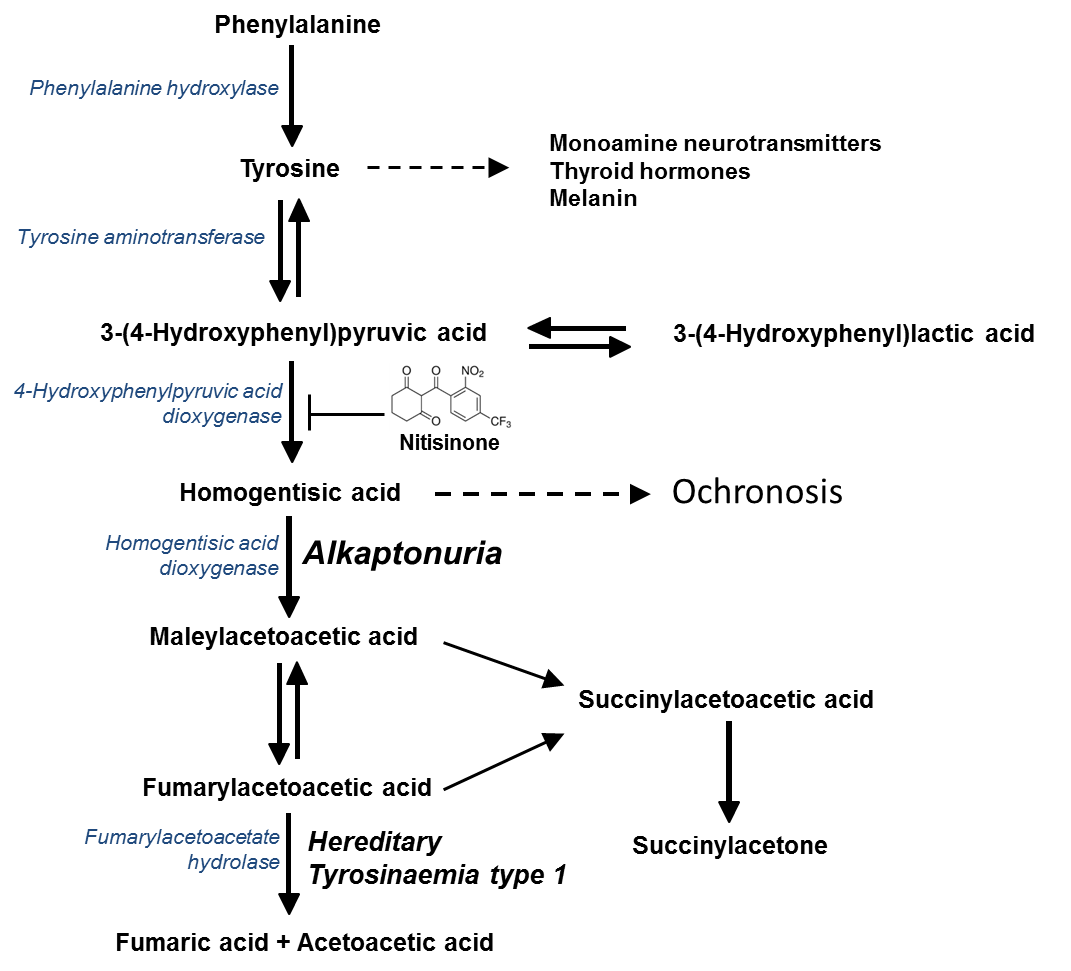


**Figure S1.** Tyrosine metabolic pathway – highlighting (i) the metabolic fate of tyrosine in health, (ii) site of the enzyme defect observed in Alkaptonuria, homogentisate dioxygenase (HGD, EC 1.13.11.5) and Hereditary Tyrosinaemia type-1, fumarylacetoacetate hydrolase (FAH, EC 3.7.1.2), and (iii) the site where nitisinone inhibits 4-hydroxyphenylpyruvate dioxygenase (HPPD, EC 1.13.11.27) activity.
