# Supplementary material for: Evaluation of the serum metabolome of patients with alkaptonuria before and after two years of treatment with nitisinone using LC‐QTOF‐MS
Source: JIMD Rep. 2019 May 31;48(1):67–74. doi: 10.1002/jmd2.12042 (PMC6606987; doi:10.1002/jmd2.12042)
Supplement: Supplementary file 3 — Table S1 Summary of accurate mass and retention time data for metabolites that make up in‐house AMRT database used in this study. [file JMD2-48-67-s003.docx]

**Table S1.** Summary of accurate mass and retention time data for metabolites that make up in-house AMRT database used in this study.

This database is adapted from a previous publication (<https://doi.org/10.6084/m9.figshare.c.4378235>). *The accurate mass and retention time of compounds in bold italics are based on elution time associated with theoretical monoisotopic mass.

| **Metabolite name** | **Empirical Formula** | **Neutral Monoisotopic Mass** | **Retention time (mins)** |
| --- | --- | --- | --- |
| (2-AMINOETHYL)PHOSPHONATE | C2H8NO3P | 125.0242 | 1.25 |
| (R)-MALATE | C4H6O5 | 134.0215 | 1.58 |
| (R,R)-TARTARIC ACID | C4H6O6 | 150.0164 | 1.46 |
| (S)-DIHYDROOROTATE | C5H6N2O4 | 158.0328 | 1.61 |
| (S)-MALATE | C4H6O5 | 134.0215 | 1.58 |
| (S,S)-TARTARIC ACID | C4H6O6 | 150.0164 | 1.46 |
| 1,2-DIDECANOYL-SN-GLYCERO-3-PHOSPHOCHOLINE | C28H56NO8P | 565.3744 | 13.32 |
| 10-HYDROXYDECANOATE | C10H20O3 | 188.1412 | 9.27 |
| 1-AMINOCYCLOPROPANE-1-CARBOXYLATE | C4H7NO2 | 101.0477 | 1.30 |
| 1-HYDROXY-2-NAPHTHOATE | C11H8O3 | 188.0473 | 9.90 |
| 1-METHYL-6,7-DIHYDROXY-1,2,3,4-TETRAHYDROISOQUINOLINE | C10H13NO2 | 179.0946 | 2.15 |
| 1-METHYLADENOSINE | C11H15N5O4 | 281.1124 | 4.39 |
| 1-NAPHTHYLAMINE | C10H9N | 143.0735 | 10.71 |
| 1-OLEOYL-RAC-GLYCEROL | C21H40O4 | 356.2927 | 14.02 |
| 2,3-DIHYDROXYBENZOATE | C7H6O4 | 154.0266 | 6.26 |
| 2,4-DIHYDROXYACETOPHENONE | C8H8O3 | 152.0473 | 3.22 |
| 2,4-DIHYDROXYPTERIDINE | C6H4N4O2 | 164.0334 | 2.86 |
| 2,4-DIHYDROXYPYRIMIDINE-5-CARBOXYLIC ACID | C5H4N2O4 | 156.0171 | 2.22 |
| 2,5-DIHYDROXYBENZOATE | C7H6O4 | 154.0266 | 4.67 |
| 2,5-DIMETHYLPYRAZINE | C6H8N2 | 108.0687 | 9.74 |
| 2-AMINO-2-METHYLPROPANOATE | C4H9NO2 | 103.0633 | 1.35 |
| 2-AMINOPHENOL | C6H7NO | 109.0528 | 2.16 |
| 2-DEOXYADENOSINE | C10H13N5O3 | 251.1018 | 3.40 |
| 2-DEOXYCYTIDINE 5-MONOPHOSPHATE | C9H14N3O7P | 307.0569 | 1.57 |
| 2-DEOXY-D-GLUCOSE | C6H12O5 | 164.0685 | 1.43 |
| 2-DEOXYGUANOSINE | C10H13N5O4 | 267.0968 | 3.22 |
| 2-DEOXYGUANOSINE 5-MONOPHOSPHATE | C10H14N5O7P | 347.0631 | 2.21 |
| 2-DEOXYURIDINE 5-MONO-PHOS-PHATE | C9H13N2O8P | 308.0410 | 2.04 |
| 2-HYDROXY-4-(METHYLTHIO)BUTYRIC ACID | C5H10O3S | 150.0351 | 4.92 |
| 2-HYDROXYBUTYRIC ACID | C4H8O3 | 104.0473 | 3.13 |
| 2-HYDROXYPHENYLACETIC ACID | C8H8O3 | 152.0473 | 6.00 |
| 2-HYDROXYPYRIDINE | C5H5NO | 95.0371 | 3.11 |
| 2-METHYLGLUTARIC ACID | C6H10O4 | 146.0579 | 4.74 |
| 2-METHYLMALEATE | C5H6O4 | 130.0266 | 2.84 |
| 2-OXOADIPATE | C6H8O5 | 160.0372 | 2.08 |
| 2-QUINOLINECARBOXYLIC ACID | C10H7NO2 | 173.0477 | 5.75 |
| 3-(2-HYDROXYPHENYL)PROPANOATE | C9H10O3 | 166.0630 | 7.09 |
| 3-(4-HYDROXYPHENYL)LACTATE | C9H10O4 | 182.0579 | 4.75 |
| 3,3,5-TRIIODOTHYRONINE | C15H12I3NO4 | 650.7900 | 9.38 |
| 3,4-DIHYDROXYBENZOATE | C7H6O4 | 154.0266 | 4.31 |
| 3,4-DIHYDROXY-L-PHENYLALANINE | C9H11NO4 | 197.0688 | 1.77 |
| 3,4-DIHYDROXYPHENYL GLYCOL | C8H10O4 | 170.0579 | 2.23 |
| 3,4-DIHYDROXYPHENYLACETATE | C8H8O4 | 168.0423 | 4.43 |
| 3,5-CYCLIC AMP | C10H12N5O6P | 329.0525 | 3.47 |
| 3,5-DIIODO-L-THYRONINE | C15H13I2NO4 | 524.8934 | 8.32 |
| 3,5-DIIODO-L-TYROSINE | C9H9I2NO3 | 432.8672 | 6.17 |
| 3-ALPHA,11-BETA,17-ALPHA,21-TETRAHYDROXY- 5-ALPHA-PREGNAN-20-ONE | C21H34O5 | 366.2406 | 9.17 |
| 3ALPHA,12ALPHA-DIHYDROXY-5BETA-CHOLANATE | C24H40O4 | 392.2927 | 13.74 |
| 3ALPHA-HYDROXY-5BETA-CHOLANATE | C24H40O3 | 376.2977 | 13.73 |
| 3-AMINO-4-HYDROXYBENZOIC ACID | C7H7NO3 | 153.0426 | 2.43 |
| 3-AMINO-5-HYDROXYBENZOIC ACID | C7H7NO3 | 153.0426 | 2.43 |
| 3-AMINOISOBUTANOATE | C4H9NO2 | 103.0633 | 1.29 |
| 3-DEHYDROSHIKIMATE | C7H8O5 | 172.0372 | 1.82 |
| 3-HYDROXY-3-METHYLGLUTARATE | C6H10O5 | 162.0528 | 2.89 |
| 3-HYDROXYANTHRANILATE | C7H7NO3 | 153.0426 | 4.62 |
| 3-HYDROXYBENZALDEHYDE | C7H6O2 | 122.0368 | 6.10 |
| 3-HYDROXYBENZOATE | C7H6O3 | 138.0317 | 5.70 |
| 3-HYDROXYBENZYL ALCOHOL | C7H8O2 | 124.0524 | 4.70 |
| 3-HYDROXYBUTANOIC ACID | C4H8O3 | 104.0473 | 5.77 |
| 3-HYDROXYKYNURENINE | C10H12N2O4 | 224.0797 | 2.19 |
| 3-HYDROXYPHENYLACETATE | C8H8O3 | 152.0473 | 5.69 |
| 3-METHOXY-4-HYDROXYMANDELATE | C9H10O5 | 198.0528 | 3.22 |
| 3-METHOXY-L-TYROSINE | C10H13NO4 | 211.0845 | 3.13 |
| 3-METHOXYTYRAMINE | C9H13NO2 | 167.0946 | 3.20 |
| 3-METHYGLUTARIC ACID | C6H10O4 | 146.0579 | 4.71 |
| 3-METHYL-2-OXINDOLE | C9H9NO | 147.0684 | 7.84 |
| 3-METHYL-2-OXOVALERIC ACID | C6H10O3 | 130.0630 | 5.09 |
| 3-METHYLADENINE | C6H7N5 | 149.0701 | 1.70 |
| 3-METHYLHISTAMINE | C6H11N3 | 125.0953 | 1.26 |
| 3-NITRO-L-TYROSINE | C9H10N2O5 | 226.0590 | 4.06 |
| 3-SULFINO-L-ALANINE | C3H7NO4S | 153.0096 | 1.39 |
| 3-UREIDOPROPIONATE | C4H8N2O3 | 132.0535 | 1.72 |
| 4-ACETAMIDOBUTANOATE | C6H11NO3 | 145.0739 | 3.14 |
| 4-AMINOBENZOATE | C7H7NO2 | 137.0477 | 4.41 |
| 4-COUMARATE | C9H8O3 | 164.0473 | 6.68 |
| 4-GUANIDINOBUTANOATE | C5H11N3O2 | 145.0851 | 1.49 |
| 4-HYDROXY-2-QUINOLINECARBOXYLIC ACID | C10H7NO3 | 189.0426 | 5.31 |
| 4-HYDROXY-3-METHOXYPHENYLGLYCOL | C9H12O4 | 184.0736 | 3.73 |
| 4-HYDROXYBENZALDEHYDE | C7H6O2 | 122.0368 | 5.84 |
| 4-HYDROXYBENZOATE | C7H6O3 | 138.0317 | 5.31 |
| 4-HYDROXY-L-PHENYLGLYCINE | C8H9NO3 | 167.0582 | 1.37 |
| 4-HYDROXY-L-PROLINE | C5H9NO3 | 131.0582 | 1.46 |
| 4-HYDROXYPHENYLACETATE | C8H8O3 | 152.0473 | 5.31 |
| 4-IMIDAZOLEACETIC ACID | C5H6N2O2 | 126.0429 | 1.29 |
| 4-METHYL-2-OXO-PENTANOIC ACID | C6H10O3 | 130.0630 | 5.15 |
| 4-METHYL-2-OXOVALERIC ACID | C6H10O3 | 130.0630 | 5.36 |
| 4-METHYLCATECHOL | C7H8O2 | 124.0524 | 6.41 |
| 4-PYRIDOXATE | C8H9NO4 | 183.0532 | 3.22 |
| 4-QUINOLINECARBOXYLIC ACID | C10H7NO2 | 173.0477 | 3.23 |
| 5,6-DIHYDROURACIL | C4H6N2O2 | 114.0429 | 1.32 |
| 5-AMINOIMIDAZOLE-4-CARBOXAMIDE-1-BETA-D-RIBOFURANOSYL 5-MONOPHOSPHATE | C9H15N4O8P | 338.0627 | 1.71 |
| 5-AMINOPENTANOATE | C5H11NO2 | 117.0791 | 5.33 |
| 5-CMP | C9H14N3O8P | 323.0519 | 1.59 |
| 5-DEOXYADENOSINE | C10H13N5O3 | 251.1018 | 4.13 |
| 5-HYDROXYINDOLEACETATE | C10H9NO3 | 191.0582 | 5.02 |
| 5-HYDROXY-L-TRYPTOPHAN | C11H12N2O3 | 220.0848 | 3.08 |
| 5-HYDROXYMETHYLURACIL | C5H6N2O3 | 142.0378 | 1.74 |
| 5-METHYLCYTOSINE HYDROCLORIDE | C5H7N3O | 125.0589 | 1.39 |
| 5-METHYLTHIOADENOSINE | C11H15N5O3S | 297.0896 | 5.34 |
| 5-OXO-D-PROLINE | C5H7NO3 | 129.0426 | 2.13 |
| 5-OXO-L-PROLINE | C5H7NO3 | 129.0426 | 2.12 |
| 5-VALEROLACTONE | C5H8O2 | 100.0524 | 7.14 |
| 6-CARBOXYHEXANOATE | C7H12O4 | 160.0736 | 5.90 |
| 6-DEOXY-L-GALACTOSE | C6H12O5 | 164.0685 | 1.57 |
| 6-HYDROXYNICOTINATE | C6H5NO3 | 139.0269 | 3.24 |
| ACETOACETATE | C4H6O3 | 102.0317 | 2.19 |
| ACETOIN | C4H8O2 | 88.0524 | 1.34 |
| ACETYL-TYROSINE | C9H11NO3 | 223.0844 | 4.81 |
| ADENINE | C5H5N5 | 135.0545 | 1.64 |
| ADENINE HYDROCHLORIDE HYDRATE | C5H5N5 | 135.0545 | 1.69 |
| ADENOSINE | C10H13N5O4 | 267.0968 | 3.22 |
| ADENOSINE 2,3-CYCLIC MONOPHOSPHATE | C10H12N5O6P | 329.0525 | 2.70 |
| ADENOSINE 3,5-CYCLIC MONOPHOSPHATE | C10H12N5O6P | 329.0525 | 3.47 |
| ADENOSINE 3,5-DIPHOSPHATE | C10H15N5O10P2 | 427.0294 | 1.64 |
| ADENOSINE 5-DIPHOSPHORIBOSE | C15H23N5O14P2 | 559.0717 | 1.65 |
| ADENOSINE 5-MONOPHOSPHATE | C10H14N5O7P | 347.0631 | 1.82 |
| ADENOSINE-5-DIPHOSPHOGLUCOSE | C16H25N5O15P2 | 589.0822 | 1.65 |
| ADIPIC ACID | C6H10O4 | 146.0579 | 4.62 |
| ALLANTOIN | C4H6N4O3 | 158.0440 | 1.36 |
| ALLOSE | C6H12O6 | 180.0634 | 1.35 |
| ALPHA-AMINOADIPATE | C6H11NO4 | 161.0688 | 1.38 |
| ALPHA-D-GALACTOSE 1-PHOSPHATE | C6H13O9P | 260.0297 | 1.33 |
| ALPHA-D-GLUCOSE | C6H12O6 | 180.0634 | 1.34 |
| ALPHA-D-GLUCOSE 1-PHOSPHATE | C6H13O9P | 260.0297 | 1.40 |
| ALPHA-HYDROXYISOBUTYRIC ACID | C4H8O3 | 104.0473 | 3.20 |
| ALPHA-KETOGLUTARIC ACID | C5H6O5 | 146.0215 | 1.67 |
| AMYLOSE | C14H26O11 | 370.1475 | 10.28 |
| ANILINE | C6H7N | 93.0578 | 7.33 |
| ANTHRANILATE | C7H7NO2 | 137.0477 | 6.21 |
| ARABINOSE | C5H10O5 | 150.0528 | 1.38 |
| ARACHIDIC ACID | C20H40O2 | 312.3028 | 14.30 |
| ASCORBATE | C6H8O6 | 176.0321 | 1.61 |
| AZELAIC ACID | C9H16O4 | 188.1049 | 8.12 |
| BENZALDEHYDE | C7H6O | 106.0419 | 9.37 |
| BENZOIC ACID | C7H6O2 | 122.0368 | 7.54 |
| BENZYLAMINE | C7H9N | 107.0735 | 3.05 |
| BETA-ALANINE | C3H7NO2 | 89.0477 | 1.24 |
| BETA-CAROTENE | C40H56 | 536.4382 | 13.81 |
| BETAINE | C5H11NO2 | 117.0790 | 1.33 |
| BILIVERDIN | C33H34N4O6 | 582.2478 | 11.34 |
| BIOTIN | C10H16N2O3S | 244.0882 | 5.98 |
| BIS(2-ETHYLHEXYL)PHTHALATE | C24H38O4 | 390.2770 | 13.51 |
| BIS(3-AMINOPROPYL)AMINE | C6H17N3 | 131.1422 | 1.41 |
| BUTANAL | C4H8O | 72.0575 | 8.76 |
| CAFFEATE | C9H8O4 | 180.0423 | 6.17 |
| CAFFEINE | C8H10N4O2 | 194.0804 | 5.97 |
| CATECHOL | C6H6O2 | 110.0368 | 4.70 |
| CHENODEOXYCHOLATE | C24H40O4 | 392.2927 | 12.20 |
| CHOLATE | C24H40O5 | 408.2876 | 11.61 |
| CHOLESTERYL ACETATE | C29H48O2 | 428.3654 | 12.49 |
| CIS-4-HYDROXY-D-PROLINE | C5H9NO3 | 131.0582 | 1.29 |
| CITRAMALATE | C5H8O5 | 148.0372 | 2.41 |
| CITRATE | C6H8O7 | 192.0270 | 1.92 |
| CITRULLINE | C6H13N3O3 | 175.0957 | 1.29 |
| CORTICOSTERONE | C21H30O4 | 346.2144 | 9.90 |
| CORTISOL | C21H30O5 | 362.2093 | 9.27 |
| CORTISOL 21-ACETATE | C23H32O6 | 404.2199 | 9.98 |
| CREATINE | C4H9N3O2 | 131.0695 | 1.32 |
| CREATINE PHOSPHATE | C4H10N3O5P | 211.0358 | 1.42 |
| CREATININE | C4H7N3O | 113.0589 | 1.21 |
| CYCLOPENTANONE | C5H8O | 84.0575 | 8.16 |
| CYTIDINE | C9H13N3O5 | 243.0855 | 1.45 |
| CYTIDINE 2,3-CYCLIC MONO-PHOS-PHATE | C9H12N3O7P | 305.0413 | 1.48 |
| CYTIDINE 5-DIPHOSPHOCHOLINE | C14H26N4O11P2 | 488.1073 | 1.32 |
| CYTOSINE | C4H5N3O | 111.0433 | 1.44 |
| D-(-)-3-PHOSPHOGLYCERIC ACID | C3H7O7P | 185.9929 | 1.57 |
| D-(-)-ARABINOSE | C5H10O5 | 150.0528 | 1.37 |
| D-(+)-CELLOBIOSE | C12H22O11 | 342.1162 | 1.39 |
| D-(+)-GALACTURONIC ACID | C6H10O7 | 194.0427 | 1.34 |
| D-(+)-RAFFINOSE | C18H32O16 | 504.1690 | 1.45 |
| D-(+)-TREHALOSE | C12H22O11 | 342.1162 | 1.37 |
| D-ALANINE | C3H7NO2 | 89.0477 | 1.42 |
| DAMP | C10H14N5O6P | 331.0682 | 2.19 |
| D-ASPARTATE | C4H7NO4 | 133.0375 | 1.28 |
| DEHYDROASCORBATE | C6H6O6 | 174.0164 | 1.51 |
| DEHYDRO-L-(+)-ASCORBIC ACID DIMER | C6H6O6 | 174.0164 | 1.37 |
| DEOXYCARNITINE | C7H15NO2 | 145.1103 | 1.30 |
| DEOXYCHOLATE | C24H40O4 | 392.2927 | 12.29 |
| DEOXYCORTICOSTERONE ACETATE | C23H32O4 | 372.2301 | 11.26 |
| DEOXYCYTIDINE | C9H13N3O4 | 227.0906 | 1.58 |
| DEOXYRIBOSE | C5H10O4 | 134.0579 | 8.91 |
| DETHIOBIOTIN | C10H18N2O3 | 214.1317 | 6.93 |
| D-FRUCTOSE 6-PHOSPHATE | C6H13O9P | 260.0297 | 1.40 |
| D-GALACTOSE | C6H12O6 | 180.0634 | 1.33 |
| D-GLUCONATE | C6H12O7 | 196.0583 | 1.36 |
| D-GLUCONO-1,5-LACTONE | C6H12O7 | 196.0583 | 1.36 |
| D-GLUCOSAMINE 6-PHOSPHATE | C6H14NO8P | 259.0457 | 1.22 |
| D-GLUCOSE 6-PHOSPHATE | C6H13O9P | 260.0297 | 1.39 |
| D-GLUCURONIC ACID | C6H10O7 | 194.0427 | 1.33 |
| D-GLUCURONOLACTONE | C6H10O7 | 194.0427 | 1.32 |
| D-GLYCERIC ACID | C3H6O4 | 106.0266 | 1.46 |
| D-GULONIC ACID GAMA-LACTONE | C6H12O7 | 196.0583 | 1.35 |
| DIACETYL | C4H6O2 | 86.0368 | 9.48 |
| DIHYDROFOLATE | C19H21N7O6 | 443.1553 | 5.33 |
| DIHYDROXYACETONE PHOSPHATE | C3H7O6P | 169.9980 | 1.43 |
| DIHYDROXYMANDELIC ACID | C8H8O5 | 184.0372 | 1.94 |
| DIMETHYLBENZIMIDAZOLE | C9H10N2 | 146.0844 | 6.05 |
| D-LACTOSE | C12H22O11 | 342.1162 | 1.33 |
| DL-KYNEURENINE | C10H12N2O3 | 208.0848 | 3.51 |
| DL-NORMETANEPHRINE | C9H13NO3 | 183.0895 | 1.61 |
| D-LYXOSE | C5H10O5 | 150.0528 | 1.37 |
| D-MANNOSAMINE | C6H13NO5 | 179.0794 | 1.27 |
| D-MANNOSE 6-PHOSPHATE | C6H13O9P | 260.0297 | 1.32 |
| DOCOSAHEXAENOIC ACID | C22H32O2 | 328.2402 | 13.29 |
| DOPAMINE | C8H11NO2 | 153.0790 | 1.66 |
| D-PANTOTHENIC ACID | C9H17NO5 | 219.1107 | 4.34 |
| D-PSICOSE | C6H12O6 | 180.0634 | 1.40 |
| D-RIBOSE | C5H10O5 | 150.0528 | 1.41 |
| D-RIBOSE 5-PHOSPHATE | C5H11O8P | 230.0192 | 1.41 |
| D-SACCHARIC ACID | C6H10O8 | 210.0376 | 1.42 |
| D-SORBITOL | C6H14O6 | 182.0790 | 1.35 |
| D-TAGATOSE | C6H12O6 | 180.0634 | 1.36 |
| D-TRYPTOPHAN | C11H12N2O2 | 204.0899 | 4.60 |
| D-XYLOSE | C5H10O5 | 150.0528 | 1.37 |
| ELAIDIC ACID | C18H34O2 | 282.2559 | 13.64 |
| EPINEPHRINE | C9H13NO3 | 183.0895 | 1.38 |
| ERUCIC ACID | C22H42O2 | 338.3185 | 14.35 |
| ERYTHRITOL | C4H10O4 | 122.0579 | 1.37 |
| ESTRADIOL-17ALPHA | C18H24O2 | 272.1776 | 9.97 |
| ETHYL 3-INDOLEACETATE | C12H13NO2 | 203.0946 | 9.50 |
| ETHYLMALONIC ACID | C5H8O4 | 132.0423 | 3.83 |
| FERULATE | C10H10O4 | 194.0579 | 7.04 |
| FLAVIN ADENINE DINUCLEOTIDE | C27H33N9O15P2 | 785.1571 | 5.97 |
| FOLIC ACID | C19H19N7O6 | 441.1397 | 5.43 |
| FORMYL-L-METHIONYL PEPTIDE | C6H11NO3S | 177.0460 | 4.55 |
| FUMARATE | C4H4O4 | 116.0110 | 2.12 |
| GALACTARATE | C6H10O8 | 210.0376 | 1.35 |
| GALACTITOL | C6H14O6 | 182.0790 | 1.32 |
| ***GAMMA-GLUTAMYL-TYROSINE**** | ***C14H18N2O6*** | ***310.1164*** | **3.81** |
| GAMMA-LINOLENIC ACID | C18H30O2 | 278.2246 | 13.51 |
| GLUCONIC ACID | C6H12O7 | 196.0583 | 1.37 |
| GLUCOSAMINATE | C6H13NO6 | 195.0743 | 1.25 |
| GLUTATHIONE | C10H17N3O6S | 307.0838 | 1.80 |
| GLYCERALDEHYDE | C3H6O3 | 90.0317 | 1.40 |
| GLYCERATE | C3H6O4 | 106.0266 | 2.27 |
| GLYCEROL | C3H8O3 | 92.0473 | 1.42 |
| GLYCEROL 2-PHOSPHATE | C3H9O6P | 172.0137 | 1.45 |
| GLYCERYL TRIMYRISTATE | C45H86O6 | 722.6424 | 14.64 |
| GLYCINE | C2H5NO2 | 75.0320 | 1.23 |
| GLYCOCHOLATE | C26H43NO6 | 465.3090 | 11.04 |
| GLYOXYLIC ACID | C2H2O3 | 74.0004 | 1.49 |
| GUAIACOL | C7H8O2 | 124.0524 | 10.49 |
| GUANIDINOACETATE | C3H7N3O2 | 117.0538 | 1.39 |
| GUANINE | C5H5N5O | 151.0494 | 1.72 |
| GUANOSINE | C10H13N5O5 | 283.0917 | 2.98 |
| GUANOSINE 3,5-CYCLIC MONOPHOSPHATE | C10H12N5O7P | 345.0474 | 3.46 |
| GUANOSINE 5-DIPHOSPHO-D-MANNOSE | C16H25N5O16P2 | 605.0772 | 1.75 |
| GUANOSINE 5-DIPHOSPHOGLUCOSE | C16H25N5O16P2 | 605.0772 | 1.83 |
| GUANOSINE 5-MONOPHOSPHATE | C10H14N5O8P | 363.0580 | 1.88 |
| HEPTADECANOATE | C17H34O2 | 270.2559 | 13.71 |
| HEXADECANOL | C16H34O | 242.2610 | 12.60 |
| HIPPURATE | C9H9NO3 | 179.0582 | 5.48 |
| HOMOCYSTEINE | C4H9NO2S | 135.0354 | 1.41 |
| HOMOCYSTINE | C8H16N2O4S2 | 268.0551 | 1.42 |
| HOMOGENTISATE | C8H8O4 | 168.0423 | 3.54 |
| HOMOSERINE | C4H9NO3 | 119.0582 | 1.27 |
| HOMOVANILLATE | C9H10O4 | 182.0579 | 5.89 |
| HYDROQUINONE | C6H6O2 | 110.0368 | 4.67 |
| HYDROXYPYRUVATE | C3H4O4 | 104.0110 | 1.44 |
| HYPOTAURINE | C2H7NO2S | 109.0197 | 1.28 |
| HYPOXANTHINE | C5H4N4O | 136.0385 | 2.07 |
| INDOLE-3-ACETALDEHYDE | C10H9NO | 159.0684 | 4.66 |
| INDOLE-3-ACETAMIDE | C10H10N2O | 174.0793 | 6.04 |
| INDOLE-3-ACETATE | C10H9NO2 | 175.0633 | 7.27 |
| INDOLE-3-ACETIC ACID | C10H9NO2 | 175.0633 | 7.27 |
| INDOLE-3-ETHANOL | C10H11NO | 161.0841 | 5.94 |
| ***INDOLE-3-LACTATE**** | ***C11H11NO3*** | ***205.0738*** | **6.66** |
| INDOLE-3-PYRUVIC ACID | C11H9NO3 | 203.0582 | 6.00 |
| INDOXYL SULFATE | C8H7NO4S | 213.0096 | 5.03 |
| INOSINE | C10H12N4O5 | 268.0808 | 2.96 |
| INOSINE 5-MONOPHOSPHATE | C10H13N4O8P | 348.0471 | 1.95 |
| INOSINE 5-PHOSPHATE | C10H13N4O8P | 348.0471 | 1.93 |
| ISOCITRIC ACID | C6H8O7 | 192.0270 | 1.63 |
| ITACONATE | C5H6O4 | 130.0266 | 3.44 |
| L-ALANINE | C3H7NO2 | 89.0477 | 1.26 |
| L-ALLOTHREONINE | C4H9NO3 | 119.0582 | 1.29 |
| LANOSTEROL | C30H50O | 426.3862 | 14.08 |
| L-ARABITOL | C5H12O5 | 152.0685 | 1.36 |
| L-ASPARAGINE | C4H8N2O3 | 132.0535 | 1.25 |
| L-ASPARTATE | C4H7NO4 | 133.0375 | 1.28 |
| LAURIC ACID | C12H24O2 | 200.1776 | 12.52 |
| LAUROYLCARNITINE | C19H37NO4 | 343.2723 | 10.93 |
| L-CARNITINE | C7H15NO3 | 161.1052 | 1.23 |
| L-CYSTATHIONINE | C7H14N2O4S | 222.0674 | 1.21 |
| L-CYSTEIC ACID | C3H7NO5S | 169.0045 | 1.38 |
| L-CYSTEINE | C3H7NO2S | 121.0197 | 1.35 |
| L-CYSTINE | C6H12N2O4S2 | 240.0238 | 1.23 |
| LEUCINE | C6H13NO2 | 131.0946 | 2.74 |
| L-GLUTAMIC ACID | C5H9NO4 | 147.0532 | 1.30 |
| L-GLUTAMINE | C5H10N2O3 | 146.0691 | 1.26 |
| L-HOMOCYSTEINE THIOLACTONE | C4H7NOS | 117.0248 | 1.34 |
| LINOLEATE | C18H32O2 | 280.2402 | 13.38 |
| LIPOAMIDE | C8H15NOS2 | 205.0595 | 8.16 |
| L-ISOLEUCINE | C6H13NO2 | 131.0946 | 2.55 |
| L-KYNURENINE | C10H12N2O3 | 208.0848 | 3.50 |
| L-METHIONINE | C5H11NO2S | 149.0510 | 1.85 |
| L-METHIONINE SULFOXIMINE | C5H12N2O3S | 180.0569 | 1.25 |
| L-NORVALINE | C5H11NO2 | 117.0790 | 1.71 |
| L-PHENYLALANINE | C9H11NO2 | 165.0790 | 3.88 |
| L-PIPECOLIC ACID | C6H11NO2 | 129.0790 | 1.68 |
| L-PROLINE | C5H9NO2 | 115.0633 | 1.40 |
| L-RHAMNOSE | C6H12O5 | 164.0685 | 1.47 |
| L-SERINE | C3H7NO3 | 105.0426 | 1.25 |
| L-SORBOSE | C6H12O6 | 180.0634 | 1.35 |
| L-THREONINE | C4H9NO3 | 119.0582 | 1.29 |
| L-TRYPTOPHAN | C11H12N2O2 | 204.0899 | 4.60 |
| L-TRYPTOPHANAMIDE | C11H13N3O | 203.1059 | 4.05 |
| L-TYROSINE | C9H11NO3 | 181.0739 | 2.17 |
| LUMICHROME | C12H10N4O2 | 242.0804 | 8.31 |
| L-VALINE | C5H11NO2 | 117.0790 | 1.65 |
| MALEAMATE | C4H5NO3 | 115.0269 | 1.87 |
| MALEIC ACID | C4H4O4 | 116.0110 | 2.01 |
| MALEIMIDE | C4H3NO2 | 97.0164 | 1.87 |
| MALONATE | C3H4O4 | 104.0110 | 1.71 |
| MALTOSE | C12H22O11 | 342.1162 | 1.38 |
| MANDELIC ACID | C8H8O3 | 152.0473 | 4.85 |
| MANNITOL | C6H14O6 | 182.0790 | 1.35 |
| MANNOSE | C6H12O6 | 180.0634 | 1.35 |
| MELATONIN | C13H16N2O2 | 232.1212 | 7.33 |
| MELIBIOSE | C12H22O11 | 342.1162 | 1.34 |
| MERCAPTOPYRUVATE | C3H4O3S | 119.9881 | 1.99 |
| MESO-TARTARIC ACID | C4H6O6 | 150.0164 | 1.44 |
| METHYL BETA-D-GALACTOSIDE | C7H14O6 | 194.0790 | 1.56 |
| METHYL INDOLE-3-ACETATE | C11H11NO2 | 189.0790 | 7.25 |
| METHYL JASMONATE | C13H20O3 | 224.1412 | 10.21 |
| METHYL VANILLATE | C8H8O4 | 168.0423 | 6.54 |
| METHYLGUANIDINE | C2H7N3 | 73.0640 | 1.25 |
| METHYLMALONATE | C4H6O4 | 118.0266 | 1.86 |
| MEVALOLACTONE | C6H12O4 | 148.0736 | 2.69 |
| MONO-ETHYL MALONATE | C3H4O4 | 104.0110 | 1.61 |
| MONO-METHYL GLUTARATE | C6H10O4 | 146.0579 | 5.29 |
| MONO-METHYL GLUTARATE | C6H10O4 | 146.0579 | 5.29 |
| MYO-INOSITOL | C6H12O6 | 180.0634 | 1.29 |
| MYRISTIC ACID | C14H28O2 | 228.2089 | 13.08 |
| N,N-DIMETHYL-1,4-PHENYLENEDIAMINE | C8H12N2 | 136.1000 | 2.31 |
| N6-(DELTA2-ISOPENTENYL)-ADENINE | C10H13N5 | 203.1171 | 7.91 |
| N-ACETYL-D-GALACTOSAMINE | C8H15NO6 | 221.0899 | 1.44 |
| N-ACETYL-D-GLUCOSAMINE | C8H15NO6 | 221.0899 | 1.44 |
| N-ACETYL-DL-GLUTAMIC ACID | C7H11NO5 | 189.0637 | 2.26 |
| N-ACETYL-DL-METHIONINE | C7H13NO3S | 191.0616 | 5.05 |
| N-ACETYL-DL-SERINE | C5H9NO4 | 147.0532 | 1.67 |
| N-ACETYL-D-MANNOSAMINE | C8H15NO6 | 221.0899 | 1.39 |
| N-ACETYL-D-TRYPTOPHAN | C13H14N2O3 | 246.1004 | 7.02 |
| N-ACETYLGLYCINE | C4H7NO3 | 117.0426 | 1.78 |
| N-ACETYL-L-ALANINE | C5H9NO3 | 131.0582 | 2.58 |
| N-ACETYL-L-ASPARTIC ACID | C6H9NO5 | 175.0481 | 1.89 |
| N-ACETYL-L-CYSTEINE | C5H9NO3S | 163.0303 | 3.11 |
| N-ACETYL-L-LEUCINE | C8H15NO3 | 173.1052 | 6.75 |
| N-ACETYL-L-PHENYLALANINE | C11H13NO3 | 207.0895 | 6.90 |
| N-ACETYLNEURAMINATE | C11H19NO9 | 309.1060 | 1.47 |
| N-ACETYLPUTRESCINE | C6H14N2O | 130.1106 | 1.37 |
| N-ACETYLSEROTONIN | C12H14N2O2 | 218.1055 | 5.38 |
| NAD | C21H27N7O14P2 | 663.1091 | 1.76 |
| N-ALPHA-ACETYL-L-ASPARAGINE | C6H10N2O4 | 174.0641 | 1.61 |
| NALPHA-ACETYL-L-LYSINE | C8H16N2O3 | 188.1161 | 1.37 |
| N-AMIDINO-L-ASPARTATE | C5H9N3O4 | 175.0593 | 1.33 |
| N-FORMYLGLYCINE | C3H5NO3 | 103.0269 | 1.56 |
| NICOTINAMIDE | C6H6N2O | 122.0480 | 2.39 |
| NICOTINAMIDE HYPOXANTHINE DINUCLEOTIDE | C21H26N6O15P2 | 664.0931 | 1.91 |
| NICOTINAMIDE MONONUCLEOTIDE | C11H15N2O8P | 334.0566 | 1.40 |
| NICOTINATE | C6H5NO2 | 123.0320 | 2.11 |
| N-METHYL-D-ASPARTIC ACID | C5H9NO4 | 147.0532 | 1.32 |
| N-METHYL-L-GLUTAMATE | C6H11NO4 | 161.0688 | 1.35 |
| NONANOATE | C9H18O2 | 158.1307 | 12.28 |
| NORADRENALINE | C8H11NO3 | 169.0739 | 1.23 |
| NORLEUCINE | C6H13NO2 | 131.0946 | 2.78 |
| O-ACETYL-L-SERINE | C5H9NO4 | 147.0532 | 1.43 |
| OCTOPAMINE | C8H11NO2 | 153.0790 | 1.39 |
| OLEATE | C18H34O2 | 282.2559 | 13.10 |
| OMEGA-HYDROXYDODECANOIC ACID | C12H24O3 | 216.1725 | 10.52 |
| O-PHOSPHO-DL-SERINE | C3H8NO6P | 185.0089 | 1.36 |
| O-PHOSPHO-L-SERINE | C3H8NO6P | 185.0089 | 1.36 |
| O-PHOSPHORYLETHANOLAMINE | C2H8NO4P | 141.0191 | 1.25 |
| OROTATE | C5H4N2O4 | 156.0171 | 1.75 |
| OROTIC ACID | C5H4N2O4 | 156.0171 | 1.76 |
| O-SUCCINYL-L-HOMOSERINE | C8H13NO6 | 219.0743 | 1.76 |
| PALATINOSE | C12H22O11 | 342.1162 | 1.40 |
| PALMITATE | C16H32O2 | 256.2402 | 13.52 |
| PALMITOLEIC ACID | C16H30O2 | 254.2246 | 13.20 |
| PARAXANTHINE | C7H8N4O2 | 180.0647 | 5.03 |
| PENTANOATE | C5H10O2 | 102.0681 | 9.49 |
| PETROSELINIC ACID | C18H34O2 | 282.2559 | 13.11 |
| PHENETHYLAMINE | C8H11N | 121.0891 | 4.16 |
| PHENOL | C6H6O | 94.0419 | 9.82 |
| PHENYL ACETATE | C8H8O2 | 136.0524 | 3.97 |
| PHENYLACETALDEHYDE | C8H8O | 120.0575 | 9.94 |
| PHENYLACETIC ACID | C8H8O2 | 136.0524 | 4.75 |
| PHENYLETHANOLAMINE | C8H11NO | 137.0841 | 3.02 |
| PHOSPHO(ENOL)PYRUVIC ACID | C3H5O6P | 167.9824 | 1.47 |
| PHOSPHOCHOLINE | C5H14NO4P | 183.0660 | 1.30 |
| PHOSPHONOACETATE | C2H5O5P | 139.9875 | 1.60 |
| PHYLLOQUINONE | C31H46O2 | 450.3498 | 12.56 |
| PICOLINIC ACID | C6H5NO2 | 123.0320 | 1.79 |
| PIPECOLATE | C6H11NO2 | 129.0790 | 1.69 |
| PROPENOATE | C3H4O2 | 72.0211 | 6.52 |
| PROTOPORPHYRIN | C34H34N4O4 | 562.2580 | 13.69 |
| PTERIN | C6H5N5O | 163.0494 | 2.83 |
| PURINE | C5H4N4 | 120.0436 | 2.75 |
| PYRIDINE-2,3-DICARBOXYLATE | C7H5NO4 | 167.0219 | 2.12 |
| PYRIDOXAL | C8H9NO3 | 167.0582 | 1.84 |
| PYRIDOXAL 5-PHOSPHATE | C8H10NO6P | 247.0246 | 2.76 |
| PYRIDOXINE | C8H11NO3 | 169.0739 | 1.79 |
| PYRROLE-2-CARBOXYLATE | C5H5NO2 | 111.0320 | 4.71 |
| PYRUVIC ALDEHYDE | C3H4O2 | 72.0211 | 2.20 |
| QUINATE | C7H12O6 | 192.0634 | 1.47 |
| QUINOLINE | C9H7N | 129.0578 | 6.05 |
| RAC-GLYCEROL 1-MYRISTATE | C17H34O4 | 302.2457 | 13.53 |
| REICHSTEIN'S SUBSTANCE S | C21H30O4 | 346.2144 | 9.98 |
| RESORCINOL MONOACETATE | C8H8O3 | 152.0473 | 7.94 |
| RETINOATE | C20H28O2 | 300.2089 | 13.20 |
| RETINOL | C20H30O | 286.2297 | 9.02 |
| RIBITOL | C5H12O5 | 152.0685 | 1.36 |
| RIBOFLAVIN | C17H20N4O6 | 376.1383 | 6.50 |
| ROSMARINIC ACID | C18H16O8 | 360.0845 | 7.35 |
| S-(5-ADENOSYL)-L-HOMOCYSTEINE | C14H20N6O5S | 384.1216 | 2.53 |
| SALICYLAMIDE | C7H7NO2 | 137.0477 | 6.08 |
| SALICYLATE | C7H6O3 | 138.0317 | 6.91 |
| SARCOSINE | C3H7NO2 | 89.0477 | 1.30 |
| S-CARBOXYMETHYL-L-CYSTEINE | C5H9NO4S | 179.0252 | 1.43 |
| SELENOCYSTAMINE | C4H12N2Se2 | 235.9450 | 1.91 |
| SELENOMETHIONINE | C5H11NO2Se | 191.0015 | 2.18 |
| SEROTONIN | C10H12N2O | 176.0950 | 1.56 |
| S-HEXYL-GLUTATHIONE | C16H29N3O6S | 391.1777 | 8.77 |
| SHIKIMATE | C7H10O5 | 174.0528 | 1.65 |
| SN-GLYCEROL 3-PHOSPHATE | C3H9O6P | 172.0137 | 1.43 |
| SORBATE | C6H8O2 | 112.0524 | 9.28 |
| SPERMINE | C10H26N4 | 202.2157 | 1.50 |
| SPHINGANINE | C18H39NO2 | 301.2981 | 12.21 |
| SPHINGOMYELIN | C41H83N2O6P | 730.5989 | 14.01 |
| STACHYOSE | C24H42O21 | 666.2219 | 1.39 |
| STEARATE | C18H36O2 | 284.2715 | 13.88 |
| SUBERIC ACID | C8H14O4 | 174.0892 | 7.10 |
| SUCCINIC ACID | C4H6O4 | 118.0266 | 2.39 |
| SUCROSE | C12H22O11 | 342.1162 | 1.44 |
| TAURINE | C2H7NO3S | 125.0147 | 1.27 |
| TAUROLITHOCHOLATE | C26H45NO5S | 483.3018 | 11.95 |
| THEOBROMINE | C7H8N4O2 | 180.0647 | 4.55 |
| THEOPHYLLINE | C7H8N4O2 | 180.0647 | 5.23 |
| THIAMINE MONOPHOSPHATE | C12H17N4O4PS | 344.0708 | 1.22 |
| THIAMINE PYROPHOSPHATE | C12H18N4O7P2S | 424.0371 | 1.42 |
| THIOPURINE S-METHYLETHER | C6H6N4S | 166.0313 | 6.12 |
| THIOUREA | CH4N2S | 76.0095 | 1.46 |
| THYMIDINE | C10H14N2O5 | 242.0903 | 3.96 |
| THYMIDINE 5-MONOPHOSPHATE | C10H15N2O8P | 322.0566 | 2.80 |
| THYMIDINE-5-DIPHOSPHO-ALPHA-D-GLUCOSE | C16H26N2O16P2 | 564.0758 | 2.25 |
| THYMINE | C5H6N2O2 | 126.0429 | 3.05 |
| THYROTROPIN RELEASING HORMONE | C16H22N6O4 | 362.1703 | 2.12 |
| THYROXINE | C15H11I4NO4 | 776.6867 | 10.13 |
| TRANS-4-HYDROXYPROLINE | C5H9NO3 | 131.0582 | 1.44 |
| TRANS-ACONITATE | C6H6O6 | 174.0164 | 2.55 |
| TRANS-CINNAMALDEHYDE | C9H8O2 | 148.0524 | 8.74 |
| TRANS-CINNAMATE | C9H8O2 | 148.0524 | 8.69 |
| TRANS-CYCLOHEXANE-1,2-DIOL | C6H12O2 | 116.0837 | 5.22 |
| TRIGONELLINE | C7H7NO2 | 137.0477 | 1.40 |
| TRYPTAMINE | C10H12N2 | 160.1000 | 5.90 |
| TYRAMINE | C8H11NO | 137.0841 | 2.29 |
| URACIL | C4H4N2O2 | 112.0273 | 1.81 |
| URATE | C5H4N4O3 | 168.0283 | 1.90 |
| URIDINE | C9H12N2O6 | 244.0695 | 2.26 |
| URIDINE 5-DIPHOSPHOGALACTOSE | C15H24N2O17P2 | 566.0550 | 1.64 |
| URIDINE 5-DIPHOSPHOGLUCOSE | C15H24N2O17P2 | 566.0550 | 1.66 |
| URIDINE 5-DIPHOSPHO-N-ACETYLGALACTOSAMINE | C17H27N3O17P2 | 607.0816 | 1.71 |
| URIDINE 5-DIPHOSPHO-N-ACETYLGLUCOSAMINE | C17H27N3O17P2 | 607.0816 | 1.70 |
| URIDINE-5-MONOPHOSPHATE | C9H13N2O9P | 324.0359 | 1.73 |
| UROCANATE | C6H6N2O2 | 138.0429 | 1.61 |
| XANTHINE | C5H4N4O2 | 152.0334 | 2.27 |
| XANTHOSINE | C10H12N4O6 | 284.0757 | 3.49 |
| XANTHURENIC ACID | C10H7NO4 | 205.0375 | 5.03 |
| XYLITOL | C5H12O5 | 152.0685 | 1.36 |
