# Supplementary material for: Evaluation of the serum metabolome of patients with alkaptonuria before and after two years of treatment with nitisinone using LC‐QTOF‐MS
Source: JIMD Rep. 2019 May 31;48(1):67–74. doi: 10.1002/jmd2.12042 (PMC6606987; doi:10.1002/jmd2.12042)
Supplement: Supplementary file 4 — Table S2 Summary of metabolites that were aligned and matched across all samples at baseline, and after nitisinone treatment at 12‐ and 24‐months. Metabolites were aligned using Profinder software (Build 08.00, Agilent, UK); a targeted feature extraction was used to align profiled experimental data with data in the accurate mass and retention time database containing 469 intermediary metabolites. Metabolites aligned and matched in both negative (n = 249) and positive (n = 151) polarities are detailed. Feature extraction employed a window of theoretical accurate mass ± 10 ppm and retention time ± 0.15mins. Allowed species were: H+, Na+ and NH4 + for positive polarity; and H− and CHO2 − for negative polarity. Dimers were allowed for both polarities. Charge state range was 1–2. [file JMD2-48-67-s004.docx]

**Table S2.** Summary of metabolites that were aligned and matched across all samples at baseline, and after nitisinone treatment at 12- and 24-months. Metabolites were aligned using Profinder software (Build 08.00, Agilent, UK); a targeted feature extraction was used to align profiled experimental data with data in the accurate mass and retention time database containing 469 intermediary metabolites. Metabolites aligned and matched in both negative (n=249) and positive (n=151) polarities are detailed. Feature extraction employed a window of theoretical accurate mass ±10ppm and retention time ±0.15mins. Allowed species were: H^+^, Na^+^ and NH_4_^+^ for positive polarity; and H^-^ and CHO_2_^-^ for negative polarity. Dimers were allowed for both polarities. Charge state range was 1-2.

| **Compound matched to AMRT** | **Polarity** |
| --- | --- |
| (R)-MALATE | Positive |
| (S)-MALATE | Positive |
| 1-AMINOCYCLOPROPANE-1-CARBOXYLATE | Positive |
| 1-METHYLADENOSINE | Positive |
| 2-DEOXY-D-GLUCOSE | Positive |
| 2-METHYLGLUTARIC ACID | Positive |
| 2-QUINOLINECARBOXYLIC ACID | Positive |
| 3-(4-HYDROXYPHENYL)LACTATE | Positive |
| 3,4-DIHYDROXYPHENYLACETATE | Positive |
| 3ALPHA,12ALPHA-DIHYDROXY-5BETA-CHOLANATE | Positive |
| 3-METHOXY-L-TYROSINE | Positive |
| 3-METHOXYTYRAMINE | Positive |
| 3-METHYGLUTARIC ACID | Positive |
| 3-UREIDOPROPIONATE | Positive |
| 4-HYDROXY-2-QUINOLINECARBOXYLIC ACID | Positive |
| 4-HYDROXYBENZALDEHYDE | Positive |
| 4-HYDROXY-L-PROLINE | Positive |
| 4-PYRIDOXATE | Positive |
| 5-AMINOLEVULINIC ACID | Positive |
| 5-HYDROXY-L-TRYPTOPHAN | Positive |
| 5-METHYLTHIOADENOSINE | Positive |
| 5-OXO-D-PROLINE | Positive |
| 5-OXO-L-PROLINE | Positive |
| 6-CARBOXYHEXANOATE | Positive |
| 6-DEOXY-L-GALACTOSE | Positive |
| ACETYL-TYROSINE | Positive |
| ALLOSE | Positive |
| ALPHA-AMINOADIPATE | Positive |
| ALPHA-D-GALACTOSE 1-PHOSPHATE | Positive |
| ALPHA-D-GLUCOSE | Positive |
| ALPHA-D-GLUCOSE 1-PHOSPHATE | Positive |
| ALPHA-KETOGLUTARIC ACID | Positive |
| AZELAIC ACID | Positive |
| BETAINE | Positive |
| BIS(2-ETHYLHEXYL)PHTHALATE | Positive |
| CAFFEINE | Positive |
| CHOLESTERYL ACETATE | Positive |
| CIS-4-HYDROXY-D-PROLINE | Positive |
| CITRATE | Positive |
| CITRULLINE | Positive |
| CORTISOL | Positive |
| CREATINE | Positive |
| CREATININE | Positive |
| D-(+)-GALACTOSAMINE | Positive |
| D-(+)-GALACTURONIC ACID | Positive |
| D-(+)-GLUCOSAMINE | Positive |
| DEHYDROASCORBATE | Positive |
| DEOXYCARNITINE | Positive |
| D-FRUCTOSE 6-PHOSPHATE | Positive |
| D-GALACTOSE | Positive |
| D-GLUCOSE 6-PHOSPHATE | Positive |
| D-GLUCURONIC ACID | Positive |
| D-GLUCURONOLACTONE | Positive |
| DIACETYL | Positive |
| DL-KYNEURENINE | Positive |
| D-MANNOSAMINE | Positive |
| D-MANNOSE 6-PHOSPHATE | Positive |
| D-ORNITHINE | Positive |
| D-PANTOTHENIC ACID | Positive |
| D-PSICOSE | Positive |
| D-SORBITOL | Positive |
| D-TAGATOSE | Positive |
| D-TRYPTOPHAN | Positive |
| EPINEPHRINE | Positive |
| ERYTHRITOL | Positive |
| GALACTITOL | Positive |
| GAMMA-GLUTAMYL-TYROSINE | Positive |
| GLYCERALDEHYDE | Positive |
| GLYCEROL | Positive |
| GLYCEROL 2-PHOSPHATE | Positive |
| GLYCINE | Positive |
| GLYCOCHOLATE | Positive |
| GUANINE | Positive |
| HIPPURATE | Positive |
| HOMOCYSTINE | Positive |
| HOMOSERINE | Positive |
| HOMOVANILLATE | Positive |
| HYPOTAURINE | Positive |
| HYPOXANTHINE | Positive |
| INDOLE-3-ACETATE | Positive |
| INDOLE-3-ACETIC ACID | Positive |
| INDOLE-3-LACTATE | Positive |
| INOSINE | Positive |
| ISOCITRIC ACID | Positive |
| L-ALLOTHREONINE | Positive |
| L-ARABITOL | Positive |
| L-ARGININE | Positive |
| L-ASPARAGINE | Positive |
| L-CARNITINE | Positive |
| L-CYSTATHIONINE | Positive |
| L-CYSTINE | Positive |
| LEUCINE | Positive |
| L-GLUTAMIC ACID | Positive |
| L-GLUTAMINE | Positive |
| L-HISTIDINE | Positive |
| L-ISOLEUCINE | Positive |
| L-KYNURENINE | Positive |
| L-LYSINE | Positive |
| L-LYSINE MONOHYDROCHLORIDE | Positive |
| L-METHIONINE | Positive |
| L-NORVALINE | Positive |
| L-ORNITHINE | Positive |
| L-PHENYLALANINE | Positive |
| L-PIPECOLIC ACID | Positive |
| L-PROLINE | Positive |
| L-RHAMNOSE | Positive |
| L-SERINE | Positive |
| L-SORBOSE | Positive |
| L-THREONINE | Positive |
| L-TRYPTOPHAN | Positive |
| L-TYROSINE | Positive |
| L-VALINE | Positive |
| MALEIC ACID | Positive |
| MANDELIC ACID | Positive |
| MANNITOL | Positive |
| MANNOSE | Positive |
| METHYL JASMONATE | Positive |
| MYO-INOSITOL | Positive |
| N(PAI)-METHYL-L-HISTIDINE | Positive |
| N-ACETYL-DL-GLUTAMIC ACID | Positive |
| N-ACETYL-DL-METHIONINE | Positive |
| N-ACETYL-DL-SERINE | Positive |
| N-ACETYLGLYCINE | Positive |
| N-ACETYL-L-ALANINE | Positive |
| N-ACETYL-L-ASPARTIC ACID | Positive |
| N-ACETYLNEURAMINATE | Positive |
| NICOTINAMIDE | Positive |
| N-METHYL-D-ASPARTIC ACID | Positive |
| NORLEUCINE | Positive |
| O-ACETYL-L-SERINE | Positive |
| PARAXANTHINE | Positive |
| PHOSPHOCHOLINE | Positive |
| PIPECOLATE | Positive |
| PYRIDOXAL | Positive |
| PYRIDOXAMINE | Positive |
| RIBITOL | Positive |
| SN-GLYCEROL 3-PHOSPHATE | Positive |
| SPERMIDINE | Positive |
| SPHINGOMYELIN | Positive |
| SUBERIC ACID | Positive |
| TAURINE | Positive |
| THEOBROMINE | Positive |
| THEOPHYLLINE | Positive |
| TRANS-4-HYDROXYPROLINE | Positive |
| TRIGONELLINE | Positive |
| URACIL | Positive |
| URATE | Positive |
| URIDINE | Positive |
| UROCANATE | Positive |
| XANTHINE | Positive |
| XYLITOL | Positive |
| (R)-MALATE | Negative |
| (S)-MALATE | Negative |
| 1,2-DIDECANOYL-SN-GLYCERO-3-PHOSPHOCHOLINE | Negative |
| 1-AMINOCYCLOPROPANE-1-CARBOXYLATE | Negative |
| 1-METHYLADENOSINE | Negative |
| 1-METHYLNICOTINAMIDE | Negative |
| 2-AMINO-2-METHYLPROPANOATE | Negative |
| 2-DEOXY-D-GLUCOSE | Negative |
| 2-DEOXYURIDINE 5-MONO-PHOS-PHATE | Negative |
| 2-HYDROXYBUTYRIC ACID | Negative |
| 3-(4-HYDROXYPHENYL)LACTATE | Negative |
| 3ALPHA,12ALPHA-DIHYDROXY-5BETA-CHOLANATE | Negative |
| 3-AMINO-4-HYDROXYBENZOIC ACID | Negative |
| 3-AMINO-5-HYDROXYBENZOIC ACID | Negative |
| 3-AMINOISOBUTANOATE | Negative |
| 3-DEHYDROSHIKIMATE | Negative |
| 3-HYDROXYKYNURENINE | Negative |
| 3-METHOXY-L-TYROSINE | Negative |
| 3-METHOXYTYRAMINE | Negative |
| 3-METHYLADENINE | Negative |
| 3-METHYLHISTAMINE | Negative |
| 3-SULFINO-L-ALANINE | Negative |
| 3-UREIDOPROPIONATE | Negative |
| 4-AMINOBUTANOATE | Negative |
| 4-HYDROXY-3-METHOXYPHENYLGLYCOL | Negative |
| 4-HYDROXY-L-PROLINE | Negative |
| 4-HYDROXYPHENYLACETATE | Negative |
| 4-IMIDAZOLEACETIC ACID | Negative |
| 4-METHYL-2-OXO-PENTANOIC ACID | Negative |
| 4-PYRIDOXATE | Negative |
| 4-QUINOLINECARBOXYLIC ACID | Negative |
| 5-AMINOLEVULINIC ACID | Negative |
| 5-HYDROXYINDOLEACETATE | Negative |
| 5-HYDROXY-L-TRYPTOPHAN | Negative |
| 5-METHYLTHIOADENOSINE | Negative |
| 5-OXO-D-PROLINE | Negative |
| 5-OXO-L-PROLINE | Negative |
| 6-DEOXY-L-GALACTOSE | Negative |
| ADENINE | Negative |
| ADENINE HYDROCHLORIDE HYDRATE | Negative |
| ADENOSINE 5-MONOPHOSPHATE | Negative |
| AGMATINE SULFATE | Negative |
| ALLANTOIN | Negative |
| ALLOSE | Negative |
| ALPHA-AMINOADIPATE | Negative |
| ALPHA-D-GALACTOSE 1-PHOSPHATE | Negative |
| ALPHA-D-GLUCOSE | Negative |
| ALPHA-D-GLUCOSE 1-PHOSPHATE | Negative |
| ALPHA-KETOGLUTARIC ACID | Negative |
| ARABINOSE | Negative |
| AZELAIC ACID | Negative |
| BENZALDEHYDE | Negative |
| BETA-ALANINE | Negative |
| BETAINE | Negative |
| BILIVERDIN | Negative |
| BIS(2-ETHYLHEXYL)PHTHALATE | Negative |
| CAFFEINE | Negative |
| CARNOSINE | Negative |
| CHENODEOXYCHOLATE | Negative |
| CHOLESTERYL ACETATE | Negative |
| CIS-4-HYDROXY-D-PROLINE | Negative |
| CITRULLINE | Negative |
| CORTICOSTERONE | Negative |
| CORTISOL | Negative |
| CORTISOL 21-ACETATE | Negative |
| CREATINE | Negative |
| CREATININE | Negative |
| CYTIDINE | Negative |
| CYTIDINE 5-DIPHOSPHOCHOLINE | Negative |
| D-(-)-ARABINOSE | Negative |
| D-(+)-CELLOBIOSE | Negative |
| D-(+)-GALACTOSAMINE | Negative |
| D-(+)-GALACTURONIC ACID | Negative |
| D-(+)-GLUCOSAMINE | Negative |
| D-(+)-TREHALOSE | Negative |
| D-ALANINE | Negative |
| D-ASPARTATE | Negative |
| DEOXYCARNITINE | Negative |
| DEOXYCHOLATE | Negative |
| DEOXYCYTIDINE | Negative |
| D-FRUCTOSE 6-PHOSPHATE | Negative |
| D-GALACTOSE | Negative |
| D-GLUCONATE | Negative |
| D-GLUCONO-1,5-LACTONE | Negative |
| D-GLUCOSAMINE 6-PHOSPHATE | Negative |
| D-GLUCOSE 6-PHOSPHATE | Negative |
| D-GLUCURONIC ACID | Negative |
| D-GLUCURONOLACTONE | Negative |
| D-GLYCERIC ACID | Negative |
| D-GULONIC ACID GAMA-LACTONE | Negative |
| DIACETYL | Negative |
| DIETHANOLAMINE | Negative |
| D-LACTOSE | Negative |
| DL-KYNEURENINE | Negative |
| DL-NORMETANEPHRINE | Negative |
| D-LYXOSE | Negative |
| D-MANNOSAMINE | Negative |
| D-MANNOSE 6-PHOSPHATE | Negative |
| DOCOSAHEXAENOIC ACID | Negative |
| D-ORNITHINE | Negative |
| D-PANTOTHENIC ACID | Negative |
| D-PSICOSE | Negative |
| D-RIBOSE | Negative |
| D-RIBOSE 5-PHOSPHATE | Negative |
| D-SORBITOL | Negative |
| D-TAGATOSE | Negative |
| D-TRYPTOPHAN | Negative |
| D-XYLOSE | Negative |
| EPINEPHRINE | Negative |
| ERYTHRITOL | Negative |
| ETHYLMALONIC ACID | Negative |
| GALACTITOL | Negative |
| GAMMA-LINOLENIC ACID | Negative |
| GLUCONIC ACID | Negative |
| GLUCOSAMINATE | Negative |
| GLYCERALDEHYDE | Negative |
| GLYCEROL | Negative |
| GLYCEROL 2-PHOSPHATE | Negative |
| GLYCERYL TRIMYRISTATE | Negative |
| GLYCINE | Negative |
| GLYCOCHOLATE | Negative |
| GUANIDINOACETATE | Negative |
| GUANINE | Negative |
| GUANOSINE | Negative |
| HEXADECANOL | Negative |
| HIPPURATE | Negative |
| HOMOCYSTEINE | Negative |
| HOMOCYSTINE | Negative |
| HOMOGENTISATE | Negative |
| HOMOSERINE | Negative |
| HOMOVANILLATE | Negative |
| HYPOTAURINE | Negative |
| HYPOXANTHINE | Negative |
| INDOLE-3-ACETALDEHYDE | Negative |
| INDOLE-3-ACETATE | Negative |
| INDOLE-3-ACETIC ACID | Negative |
| INDOLE-3-PYRUVIC ACID | Negative |
| INOSINE | Negative |
| ISOCITRIC ACID | Negative |
| L-ALANINE | Negative |
| L-ALLOTHREONINE | Negative |
| L-ARABITOL | Negative |
| L-ARGININE | Negative |
| L-ASPARAGINE | Negative |
| L-ASPARTATE | Negative |
| LAURIC ACID | Negative |
| LAUROYLCARNITINE | Negative |
| L-CARNITINE | Negative |
| L-CYSTINE | Negative |
| L-GLUTAMIC ACID | Negative |
| L-GLUTAMINE | Negative |
| L-HISTIDINE | Negative |
| LINOLEATE | Negative |
| L-ISOLEUCINE | Negative |
| L-KYNURENINE | Negative |
| L-LYSINE | Negative |
| L-LYSINE MONOHYDROCHLORIDE | Negative |
| L-METHIONINE | Negative |
| L-NORVALINE | Negative |
| L-ORNITHINE | Negative |
| L-PIPECOLIC ACID | Negative |
| L-PROLINE | Negative |
| L-RHAMNOSE | Negative |
| L-SERINE | Negative |
| L-SORBOSE | Negative |
| L-THREONINE | Negative |
| L-TRYPTOPHAN | Negative |
| L-TYROSINE | Negative |
| L-VALINE | Negative |
| MALEAMATE | Negative |
| MALTOSE | Negative |
| MANDELIC ACID | Negative |
| MANNITOL | Negative |
| MANNOSE | Negative |
| MELIBIOSE | Negative |
| METHYL BETA-D-GALACTOSIDE | Negative |
| METHYLMALONATE | Negative |
| MEVALOLACTONE | Negative |
| MYO-INOSITOL | Negative |
| N(PAI)-METHYL-L-HISTIDINE | Negative |
| N-ACETYL-D-GALACTOSAMINE | Negative |
| N-ACETYL-D-GLUCOSAMINE | Negative |
| N-ACETYL-DL-GLUTAMIC ACID | Negative |
| N-ACETYL-DL-METHIONINE | Negative |
| N-ACETYL-DL-SERINE | Negative |
| N-ACETYL-D-MANNOSAMINE | Negative |
| N-ACETYL-D-TRYPTOPHAN | Negative |
| N-ACETYLGLYCINE | Negative |
| N-ACETYL-L-ALANINE | Negative |
| N-ACETYL-L-ASPARTIC ACID | Negative |
| N-ACETYLNEURAMINATE | Negative |
| N-ACETYLPUTRESCINE | Negative |
| N-ACETYLSEROTONIN | Negative |
| N-ALPHA-ACETYL-L-ASPARAGINE | Negative |
| N-AMIDINO-L-ASPARTATE | Negative |
| NEPSILON,NEPSILON,NEPSILON-TRIMETHYLLYSINE | Negative |
| NICOTINAMIDE | Negative |
| NICOTINAMIDE MONONUCLEOTIDE | Negative |
| N-METHYL-D-ASPARTIC ACID | Negative |
| N-METHYL-L-GLUTAMATE | Negative |
| O-ACETYL-L-SERINE | Negative |
| OCTOPAMINE | Negative |
| OMEGA-HYDROXYDODECANOIC ACID | Negative |
| OROTATE | Negative |
| OROTIC ACID | Negative |
| PALATINOSE | Negative |
| PALMITATE | Negative |
| PALMITOLEIC ACID | Negative |
| PARAXANTHINE | Negative |
| PHENETHYLAMINE | Negative |
| PHENYLACETALDEHYDE | Negative |
| PHENYLACETIC ACID | Negative |
| PHENYLETHANOLAMINE | Negative |
| PHYLLOQUINONE | Negative |
| PIPECOLATE | Negative |
| PYRIDOXAL | Negative |
| PYRIDOXAMINE | Negative |
| QUINATE | Negative |
| REICHSTEIN?S SUBSTANCE S | Negative |
| RIBITOL | Negative |
| RIBOFLAVIN | Negative |
| S-(5-ADENOSYL)-L-METHIONINE | Negative |
| SARCOSINE | Negative |
| SEROTONIN | Negative |
| SHIKIMATE | Negative |
| SN-GLYCEROL 3-PHOSPHATE | Negative |
| SORBATE | Negative |
| SPERMIDINE | Negative |
| SPHINGANINE | Negative |
| SUBERIC ACID | Negative |
| SUCCINIC ACID | Negative |
| SUCROSE | Negative |
| TAURINE | Negative |
| THEOBROMINE | Negative |
| THEOPHYLLINE | Negative |
| THIAMINE MONOPHOSPHATE | Negative |
| THYMIDINE | Negative |
| THYMINE | Negative |
| TRANS-4-HYDROXYPROLINE | Negative |
| TRANS-ACONITATE | Negative |
| TRIGONELLINE | Negative |
| URACIL | Negative |
| URATE | Negative |
| URIDINE | Negative |
| UROCANATE | Negative |
| XANTHINE | Negative |
| XANTHOSINE | Negative |
| XANTHURENIC ACID | Negative |
| XYLITOL | Negative |
